# Computational Barthel Index: an automated tool for assessing and predicting activities of daily living among nursing home patients

**DOI:** 10.1186/s12911-020-01368-8

**Published:** 2021-01-09

**Authors:** Janusz Wojtusiak, Negin Asadzadehzanjani, Cari Levy, Farrokh Alemi, Allison E. Williams

**Affiliations:** 1 Health Informatics Program, Department of Health Administration and Policy, George Mason University, Fairfax, VA USA; 2 Department of Veterans Affairs, Denver, CO USA; 3 Department of Veterans Affairs, Bay Pines, FL USA

**Keywords:** Machine learning, Supervised learning, Gerontology, Activities of daily living

## Abstract

**Background:**

Assessment of functional ability, including activities of daily living (ADLs), is a manual process completed by skilled health professionals. In the presented research, an automated decision support tool, the Computational Barthel Index Tool (CBIT), was constructed that can automatically assess and predict probabilities of current and future ADLs based on patients’ medical history.

**Methods:**

The data used to construct the tool include the demographic information, inpatient and outpatient diagnosis codes, and reported disabilities of 181,213 residents of the Department of Veterans Affairs’ (VA) Community Living Centers. Supervised machine learning methods were applied to construct the CBIT. Temporal information about times from the first and the most recent occurrence of diagnoses was encoded. Ten-fold cross-validation was used to tune hyperparameters, and independent test sets were used to evaluate models using AUC, accuracy, recall and precision. Random forest achieved the best model quality. Models were calibrated using isotonic regression.

**Results:**

The unabridged version of CBIT uses 578 patient characteristics and achieved average AUC of 0.94 (0.93–0.95), accuracy of 0.90 (0.89–0.91), precision of 0.91 (0.89–0.92), and recall of 0.90 (0.84–0.95) when re-evaluating patients. CBIT is also capable of predicting ADLs up to one year ahead, with accuracy decreasing over time, giving average AUC of 0.77 (0.73–0.79), accuracy of 0.73 (0.69–0.80), precision of 0.74 (0.66–0.81), and recall of 0.69 (0.34–0.96). A simplified version of CBIT with 50 top patient characteristics reached performance that does not significantly differ from full CBIT.

**Conclusion:**

Discharge planners, disability application reviewers and clinicians evaluating comparative effectiveness of treatments can use CBIT to assess and predict information on functional status of patients.

## Background

Knowledge about functional abilities and their decline is important for decision making regarding care provided to patients. For example, in a study by Fried [1], it was observed that patients who were aware that they were unlikely to return to their baseline functional status were less likely to proceed with hospital treatment. It is shown that the quality of life is more important than living longer [2]. Quality of life depends on many factors, one of which is patients’ functional independence. Functional ability of nursing home patients is assessed by direct observation of a skilled nurse practitioner, which is a time consuming and costly process. The assessments are often reported using the Minimum Data Set (MDS), a standardized patient evaluation instrument collected by nurses through observing patients in consultation with other care team members. In the United States, assessment data are collected by all Medicare and Medicaid-certified nursing homes and entered in MDS Section G [3]. MDS data are typically collected every three months, or whenever a patient status changes. In contrast, similar detailed functional assessments are not routinely collected for most elderly patients outside of nursing homes. To remedy this situation, this paper examines whether functional ability can be assessed and predicted through coded data available in Electronic Health Records (EHRs) or medical claims. Specifically, the focus is on the ability to independently perform activities of daily living (ADLs). Nine out of ten functional abilities in the Barthel Index (Score) were used [4, 5] as described in the Data section. The ten items that represent the ability and level of independence in performing activities of daily living include: feeding, bathing, grooming, dressing, bowel incontinence, bladder incontinence, toilet use, transfers (bed to chair and back), mobility (walking), and stairs [6].

The ability to automatically derive and predict patients’ functional status has several important uses in clinical work and research. Firstly, it may provide a more efficient and cost-effective means of assessing functional status in groups for whom functional status is currently manually assessed. In a recent review that examined functional status quality indicators, the authors concluded that using chart reviews or patient-reports is costly and administratively burdensome [7]. Secondly, it may allow for retrospective assessment of patients’ functional status for whom evaluations have not been completed. Thirdly, it can be beneficial for patients who are typically not evaluated for the purpose of comparing care across settings. Finally, predicting functional status up to one year in the future provides a basis for an informed discussion between clinicians and patients/caregivers and may help in planning care for patients.

Previously, a set of models capable of predicting trajectories of ADL improvement or decline post-hospitalization [8], as well as sequences of functional decline were constructed [9]. The former focused on predicting if patients are likely to follow one of seven pre-defined trajectories of improvement/decline. Predictions were anchored to the time of hospital discharge and diagnoses were extracted only from inpatient records of the corresponding hospitalization. The method and tool discussed in this paper, called the Computational Barthel Index Tool (CBIT), significantly extends the previous work and is designed to allow for assessment of functional status at any arbitrary moment. The tool that allows for prediction of each ADL up to one year ahead, is based on a larger cohort of patients, and uses both inpatient and outpatient diagnoses. The name is inspired by the original Barthel Index (Score), which is a standardized tool used to evaluate activities of daily living [10]. Computational machine learning methods are used to construct the index. The presented research also extends previous work [8] by incorporating temporal information about when events happened in the patient’s medical history, which was not applicable to hospitalization-only data. Many diagnoses present in medical records correlate with the patient’s functional ability, with some of these correlations being temporary and others being permanent. For example, some surgical patients have urinary incontinence for a short period after the surgery, while amputation affects the ability to walk permanently. Thus, it is assumed that the codes present in data are time-dependent. It was shown that adding temporal information can improve the accuracy of the constructed CBIT models, as discussed later in the paper.

Prediction of functional status and disability is challenging. Researchers in many studies have attempted to automatically assess and predict functional status, including ADLs. Overall, there are three main approaches to assess and predict ADLs by (1) using specific clinical data, (2) using sensor data collected by wearable devices or smarthome environments, and (3) using patient records extracted from EHR or claims data in making assessment and predictions. Despite wide selection of published works, the research presented here is unique in the latter category as its attempts to assess and predict ADLs purely based on diagnoses and demographics present in the patient records. It should be noted that there are a number of published papers that discuss ADLs as predictors of other outcomes such as disease progression and mortality [11, 12], while the focus of this study is on predicting ADLs.

Many studies attempted to predict ADLs in a specific population, i.e., related to a disease or injury [13–15], while others are more general. In one study, machine learning (ML) methods were linked to biomedical ontologies to predict functional status [16], achieving predictive accuracy of 0.6. In another work, researchers described a logistic regression-based method to predict mortality and disability post-injury for the elderly [17] with reported R^2^ of 0.86. Tarekegn et al. developed a set of models to predict disability as a metric for frailty conditions resulting in models with F-1 scores ranging between 0.74 to 0.76 [18]. Similarly, Gobbens and van Assen examined six standard frailty indicators (gait speed, physical activity, hand grip, body mass index, and fatigue and balance) for assessing ADLs, of which only gait speed was predictive of ADL disabilities [19]; however, no actual predictive accuracy was reported. More recently, Jonkman et al., constructed logistic regression-based models from four datasets to predict decline in five ADLs [20], with the average AUC of 0.72. It is clear that the above studies reported model performances below ones reported here. However, it should be mentioned that these works were performed in different settings thus no direct comparison is meaningful. A systematic review of published works related to assessing ADLs identified several commonly used predictors, including age, cognitive functioning, depression, and hospital length of stay [21]. In the data-driven approach presented here, some of the predictors are the same as those previously reported in the literature.

Not surprisingly, several research groups focused on assessing ADLs from sensor data. Assessing ADLs selected by wearable sensors is a reasonable approach as it allows for continuous monitoring rather than a snapshot of activities evaluated by a healthcare provider [21–26]. In some studies, ambient intelligence and smarthome sensors were used to assess the ability to perform ADLs. These works rely on the use of specific sensors installed in smarthome environment that monitor movement [27, 28], as well as use of specific home devices [29–31]. Further, beyond the direct application to the elderly population, activity recognition is a well-established field with several review papers available to summarize the works [32–34].

**Fig. 1:**
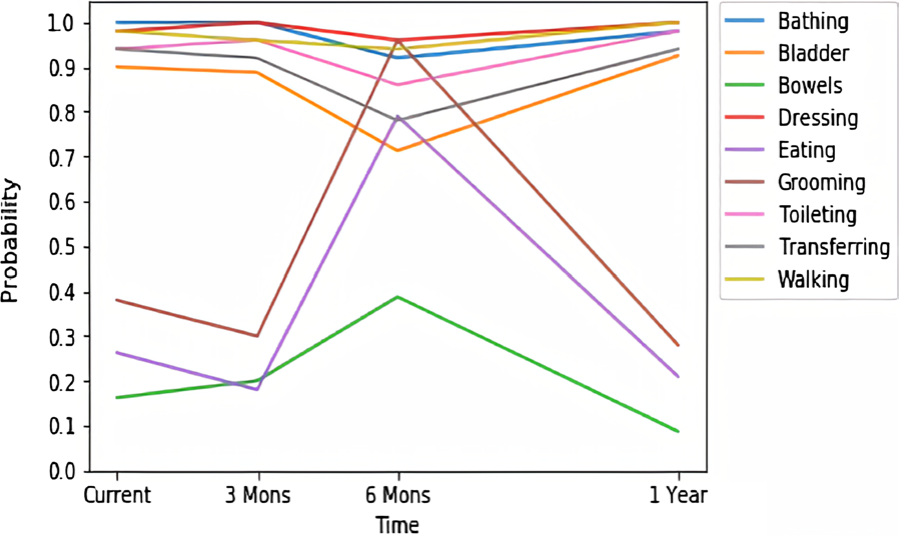
Predicted probability visualization of functional independence for a hypothetical patient up to one year ahead

The presented CBIT can be linked to an EHR through a standardized interface and used by clinicians to assess functional abilities at the time of a specific patient visit or in a batch/bulk mode to predict current functional abilities as well as ADL changes for a group of patients. The models used in the tool rely on readily available data in EHR systems or claims data and do not require additional data collection. In addition, a simplified version of the tool was developed based on 50 patient characteristics selected from amongst 578 used in the complete model. The simplified version was used to build an online calculator capable of asking limited number of questions about patients’ medical history and presenting the results in a graphical form such as exemplified in Fig. 1. In the figure, each line corresponds to one ADL plotted over time for a hypothetical patient. The horizontal axis indicates time and the vertical axis shows the probability of functional independence. It should be mentioned that this probabilistic interpretation of the prediction is not intended to indicate the level of disability, but rather the confidence the models have in predictions. In this example, the hypothetical patient is predicted to have functional independence with high probability in terms of bathing, bladder, dressing, toileting, transferring and walking. In terms of eating and grooming, this patient is predicted to temporarily recover approximately 6 months after the initial assessment and decline afterwards (see Discussion section for more details).

In the presented work, two cases are considered: when previous functional status of a patient is unknown and only diagnoses and demographics can be used as predictors, and when a patient was previously evaluated and results of that evaluation (nine previous ADL attributes) can be added to the list of predictors. Thus, two sets of models were constructed: *Evaluation models*, *M*_*E*_^*d*^_*τ*_, in which previous functional status assessment is unknown, and *Re-Evaluation models*, *M*_*RE*_^*d*^_*τ*_, in which previous functional status is known. Here *d* is an ADL (bathing, grooming, etc.), and $\tau \in \left\{ {0,90,180,365} \right\}$ is the prediction horizon (given as the number of days), i.e., how far ahead in time the value is predicted. As names suggest, *M*_*E*_^*d*^ models are used in situations in which a new patient is being evaluated in terms of ADLs, and *M*_*RE*_^*d*^* models are used *when an evaluation of the previously assessed patient needs to be refreshed as new information becomes available.

The presented research has been initiated as part of a larger IRB-approved project in the Department of Veterans Affairs (VA) with the purpose of assessing the cost and effectiveness of the Medical Foster Home program compared to traditional Community Living Centers (nursing homes) [8, 9, 35]. Determination of patients’ functional status was used as one of the characteristics to match residents in both settings for comparison purposes. In this context, the main contributions of the presented work are in (1) the development of models for assessment and prediction of ADLs up to one year ahead; (2) construction of attributes that represent time between diagnosis and prediction; (3) detailed testing and analysis of the developed models, and (4) creation of an online decision support tool.

## Methods

### Data

Data from the Department of Veterans Affairs Corporate Data Warehouse were extracted and analyzed within the VA Computing Infrastructure. The original data came from two sources: (1) medical records from the VA’s Electronic Medical Record System, and (2) MDS evaluations for nationwide VA nursing homes. Both datasets are collected as part of routine patient care and were provided to the research team in a deidentified form. The data were organized around patient evaluations using Minimum Data Set 2.0 [36], which were mapped to the nine Barthel Index categories using a previously developed procedure [8]. The Barthel Index (or Barthel Score), which measures independence in performing ADLs [4, 5] includes 10 items with the total value ranging from 0 to 100 (feeding, bathing, grooming, dressing, bowel incontinence, bladder incontinence, toilet use, transfers, mobility, and stairs). In this research, the last item of the Barthel Score (stairs) was eliminated, which was not consistently assessed and thus difficult to standardize among nursing home residents. Thus, the total considered scale is 0–90 based on the first nine items predicted independently. Each of the items in the Barthel Score has different levels of functional abilities, with highest values indicating full independence (see Additional file 1 for more details). For instance, Barthel Score captures three levels for toileting: dependent (0), needs some help (5), and independent (10). Binary output for each of the ADLs was constructed defined as fully functional vs. any level of dependency.

The data consisted of 1,901,354 MDS evaluations completed between 2000 and 2011 from which 1,151,222 complete evaluations were retrieved for 295,491 patients. The data were linked to medical records from which demographics and history of diagnoses were extracted. The EHR data are limited to services provided by the VA’s health system. The data consisted of 18,912,553 inpatient and 180,123,710 outpatient diagnosis codes using the International Classification of Diseases, ninth edition (ICD-9) standard along with corresponding dates. These codes were transformed into clinically relevant categories using Clinical Classification Software (CCS) from the Agency of Health Research and Quality (AHRQ) resulting in 281 distinct CCS codes representing health comorbidities. All diagnosis codes were combined from inpatient and outpatient records. Distinguishing between inpatient and outpatient codes is important for some applications (inpatient codes are typically treated as more severe). In the presented work, it is assumed that only information about the presence of a diagnosis along with appropriate time was important in the context of predicting disabilities, rather than distinguishing between the specific sources. Demographic information including age, race, and gender was also included. Age was recorded as a continuous variable and race was represented using one-hot vectors (0/1 values are used to indicate the presence or absence of the features). Missing data for age were imputed as mean value in the dataset and no special treatment for missing data for other attributes was needed. Patients with only one MDS evaluation were excluded to allow for modeling of change of patient status over time, resulting in a final dataset of 855,731 evaluations for 181,213 patients. The collected data were organized per MDS evaluation, resulting in the average of 4.72 ± 6.21 MDS evaluations per patient. Table 1 shows descriptive statistics of the final dataset as counted in analyzed MDS records as well as per patient, and is representative of the overall nursing home population in the VA. Most patients were male and white with an average age of over 71 years and mean Barthel Score (sum of assigned Barthel items) of about 48 out of 90, indicating overall high levels of disability in the studied population. In addition, the average score at the first evaluation was about 52. The average time between MDS evaluations was also about 100 days, which is slightly over three months.

**Table 1:** Characteristics of data

	All data		Patients with at least 2 MDS evaluations	
	MDS records	Patients	MDS records	Patients
N	1,151,222	295,491	855,731	181,213
Gender				
Male	96.8%	96.9%	96.7%	96.9%
Female	3.32%	3.1%	3.3%	3.1%
Race				
Asian	1.5%	1.4%	1.6%	1.41%
Black	13%	11.9%	13.4%	12.02%
White	58.8%	55.4%	59.9%	55.03%
Other	26.7%	31.3%	25.1%	31.53%
Age	71.89 ± 12.38	–	72.26 ± 12.31	–
Age at first MDS	–	70.8 ± 12.51	–	71.05 ± 12.43
CCS ^max^	1424.65 ± 1215.7	–	1504.17 ± 1123.78	–
CCS ^min^	619.75 ± 867.49	–	663.84 ± 889.14	–
Barthel Score	49.00 ± 29.98	–	47.81 ± 30.17	–
Score at first MDS	–	52.44 ± 29.14	–	53.6 ± 28.8
Time between	–	–	101.93 ± 234.31	143.66 ± 374.16

In addition, the distribution of values for the nine ADLs is presented in Table 2. With the exception of bladder incontinence, bowel incontinence and eating, the majority of evaluations indicate some level of dependency in performing ADLs. Lack of full independence in terms of walking is the most prominent, with 73% of evaluation records and 80% of patients. While these values are not equal to 50%, the data are reasonably balanced thus no additional resampling or balancing was required.

**Table 2:** Distribution of the nine considered ADLs

	MDS records	Patients
N	855,731	181,213
Any level of dependency		
Bathing	74.2%	77.5%
Bladder	39.7%	43.0%
Bowels	41.4%	45.3%
Dressing	66.2%	71.4%
Eating	47.8%	54.6%
Grooming	63.0%	67.1%
Toileting	60.9%	66.8%
Transferring	52.1%	60.9%
Walking	73.2%	80.1%

The numbers are proportion of data with values indicating any level of dependency

In the used data warehouse, as well as in many administrative datasets, patient medical records often span many years, making it possible to examine temporal relationships between diagnoses and the predicted events. In the presented research, a simple approach to incorporate time was used. Values of attributes corresponding to diagnoses represent time between first known occurrence of a diagnosis code and the time of MDS evaluation.

1 $$ccs_{i}^{\max } = \mathop {\max }\limits_{{t_{i} }} \left( {t_{p} - t_{i} } \right)$$

Here, (*t*_*i*_) is the time of i-th diagnosis code occurring in the data, and (*t*_*p*_) is the time of prediction. Note that each diagnosis code may be present in the data multiple times. Another set of attributes represent the last recorded occurrence of the diagnosis code relative to the time of MDS evaluation.

2 $$ccs_{i}^{\min } = \mathop {\min }\limits_{{t_{i} }} \left( {t_{p} - t_{i} } \right)$$

In the original data, diagnoses have associated dates thus days are used as unit of time. This allows counting the difference in time as the number of days. In other words $ccs_{i}^{\max }$ is the number of days separating the first occurrence of the diagnosis and the time of prediction, and $ccs_{i}^{\min }$ is the number of days separating the most recent occurrence of the diagnosis and the time of prediction.

This method of constructing attributes provides information about how long a patient suffers from a given condition as well as if the condition is still present at the time of assessment (when was the most recent diagnosis of a specific health condition). The rationale behind this approach is that for many chronic conditions that affect patients’ ability to perform ADLs over time, it is important to know how long the condition is present for the patient. Similarly, for many acute conditions, their effects on ADLs are temporary, thus only recent occurrences are important to consider. It should be noted that the chronic/acute status of a condition is not assigned ahead of time and each diagnosis is encoded using both $ccs_{i}^{\max }$ and $ccs_{i}^{\min }$. It was observed that the models tend to rank higher $ccs_{i}^{\max }$ codes for chronic conditions and $ccs_{i}^{\min }$ for acute conditions, yet full validation of this fact is out of scope of this paper.

An example of data encoded using the above method is presented in Table 3. The table shows data for two different fictitious patients. Patient 1 has two MDS evaluations in the data 90 days apart. Patient 2 also has two MDS evaluations 100 days apart. Patient 1 was diagnosed with septicemia only once, 210 days prior to the first evaluation (*ccs*_*2*_^*min*^ = *ccs*_*2*_^*max*^ = 210). The patient has not been diagnosed second time between the evaluations because both columns representing the first and most recent occurrence increased by the same amount. The patient was diagnosed with hypertension 18 days prior to the first evaluation (*ccs*_*99*_^*min*^ = 18), and for the first time 500 days prior to the first evaluation. The patient was diagnosed with hypertension again 5 days prior to the second evaluation. Similarly, Patient 2 has been diagnosed with septicemia twice, 15 and 700 days prior to the first evaluation (*ccs*_*2*_^*min*^ = *15 and ccs*_*2*_^*max*^ = 700). Patient 2 was also diagnosed with tuberculosis 71 days before the second evaluation (*ccs*_*1*_^*min*^ = *ccs*_*1*_^*max*^ = *7*1). One can also notice that Patient 1′s ADLs declined between the evaluations. Diagnoses not present/recorded in patient’s records are coded as − 999,999 and 999,999.

**Table 3:** Four example records of the data for two patients

	Demographics		ADLs		Diagnoses							
Pat	…	Age	Feed	Transferring	…	ccs_1_^min^	ccs_1_^max^	ccs_2_^min^	ccs_2_^max^	…	ccs_99_^min^	ccs_99_^max^
1	…	73	10	5		999,999	− 999,999	210	210		18	500
1	…	73	5	0		999,999	− 999,999	300	300		5	590
2	…	60	10	15		999,999	− 999,999	15	700		999,999	− 999,999
2	…	61	10	15		71	71	115	800		999,999	− 999,999

Complete data has 578 columns and 888,731 rows

Negative numbers (− 999,999) are used for coding of not present diagnoses in $ccs_{i}^{\max }$ columns because that time is intended to capture positive correlation between long-term chronic conditions and disabilities. Intuitively, the longer a patient suffers from a chronic condition (large values for time), the worse the prognosis is. When a condition is not present in the patient’s medical history, it needs to be coded as “much better” than if the patient was just diagnosed; thus, using a large negative number is reasonable. Similarly, positive numbers (999,999) are used for coding of not present diagnoses in columns, $ccs_{i}^{\min }$, because of the negative correlation of time between the most recent occurrence of conditions and disabilities. Full evaluation of this coding method in CBIT is discussed in the Results section.

### Construction of models

The presented study followed a standard experimental design used in machine learning. Patients were randomly assigned to training (90%) and testing (10%) sets. The testing set with a sufficiently large sample size (approximately 18,000 patients) was used only for final validation of the models. Training dataset was used for tenfold cross-validated hyperparameter tuning, model selection and final model construction. A selection of machine learning methods was investigated to construct models capable of assessing and predicting ADLs.

Machine learning methods are rapidly gaining popularity in medical and health applications [37] and are also applicable to the prediction of ADLs. Machine learning (ML) is an experimental field that provides a large toolset of methods that can be used for prediction. More specifically, the presented work utilizes a set of ML methods called supervised learning. These methods are intended to build models that allow for predicting outcomes for individuals based on their characteristics. The supervision comes in the form of training data in which outcomes are known for historical cases. These historical cases/patients are generalized to allow predictions for new previously unseen cases.

In the presented work, selected ML methods (regularized logistic regression, Bayesian networks, decision trees, and random forests) were evaluated in terms of their performance and it was shown that random forest stands out in terms of model quality. Random forests [38] are ensembles of decision trees (typically many), that are inferred from randomly selected subsets of data thus guaranteed to be different on sufficiently large data. Random forests are created by applying bagging (a.k.a., bootstrap aggregation) [39] to both sample and attributes (patient characteristics). Standard top-down decision tree learning algorithms are used to create individual trees. The process is repeated to create multiple trees (typically in the order of tens or hundreds). After a forest is assembled, the final classification decision is made by applying all of the trees to new examples (patients). When there is a disagreement in prediction, the trees vote on the predicted outcome. Random forests output classification scores (in the presented work, they were converted to probabilities) which in the case of the described models represent patients being disabled or functionally independent. These scores are calculated as a proportion of trees voting for a given outcome [40]. In the presented work, tenfold cross-validated hyperparameter tuning was performed. The tuning led to the selection of random forests consisting of about 100 decision trees (each model was optimized separately, and the numbers of trees were slightly different). Other algorithm parameters, including the number of randomly selected patient characteristics (number of attributes in each tree) and Gini Index [38] as an internal quality criterion were tested and set to default as they did not make any improvements.

**Fig. 2:**
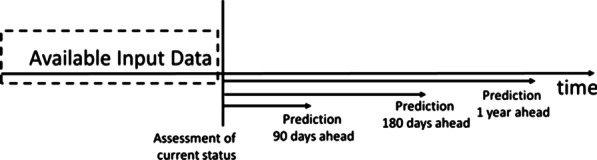
Prediction timeline. Past EHR data is used to make an assessment of current functional status as well as prediction 3, 6 months, and 1 year afterwards

The models were created to assess functional status at the time of prediction (current status), as well as to predict functional status 3, 6, and 12 months beyond the time of prediction as depicted in Fig. 2. Data available prior to the time of prediction were used to construct input attributes for the model. In the constructed models, there are 9 ADLs and 4 time points, thus there are 36 output attributes that are being predicted. Since Evaluation and Re-Evaluation models are considered separately, CBIT consists of a total of 72 models.

The quality of the constructed models was evaluated in terms of standard statistical measures used in ML, namely, accuracy (percentage of correctly predicted cases), area under the curve (AUC; often referred to as C-statistic), recall (rate of correctly identified patients with functional dependencies), precision (rate of patients with disabilities among those indicated as disabled by the model), and F1-score. Because of probabilistic interpretation of prediction results (see discussion), we consider AUC as the most important metric. Evaluation was applied on the test set of patients not being used in model construction, selection or tuning. In order to provide better insight into the created models, calibration plots (described in later section) and learning curves were also created for all developed models. The learning curves are used here to check if the amount of data used to train the models is sufficient. Curves that get flat on the right side indicate that it is unlikely that more data would improve models, while those steeply growing suggest that models could have been improved if more data were available. Learning curves for the CBIT models are available in supplemental material (See Additional files 4–7).

It should be mentioned that the presented work does not include clinical validation of the models. Also, note that the created models predict the probability of functional dependence of any level, while the graphical representation or prediction (in the web calculator discussed later and presented figures) shows the probability of functional independence. The conversion between the two is a simple operation, which is one minus probability. The reason for this conversion is that prediction of disability as a target event is conceptually cleaner from a machine learning perspective (assuming that being independent is normal, the abnormal state of disability is predicted). On the other hand, clinicians are used to having higher values represent better status (this can also be the case in in the original Barthel Index). This conversion has no effect on presented results or modeling and is only reflected in the graphical representation of results.

For the data analysis part of the project, the Microsoft SQL Server was used to preprocess data. The data preprocessing started with MDS evaluations that were later linked to other data components. Final data were analyzed using Python programming language with Scikit-learn machine learning library [41] and visualizations were done using Matplotlib Python library [42].

## Results

Computational Barthel Index Tool (CBIT) consists of a set of 72 random forest models, 36 *M*_*E*_^*d*^_*τ*_ and 36 *M*_*RE*_^*d*^_*τ*_ models. The CBIT can assess the level of functional dependency in performing ADLs and predicting functional dependency up to one year ahead by using demographics, diagnoses, and (if available) last known functional status. Table 4 presents a summary of the performance of the models for each ADL at the time of prediction, as well as 3, 6 and 12 months ahead for both *M*_*E*_^*d*^_*τ*_ and *M*_*RE*_^*d*^_*τ*_ models. The results are presented in terms of average AUC, accuracy, precision and recall of the nine outcome categories. The CBIT showed very high accuracy in assessing ADLs at a given time. The AUC of assessing if patients have any level of ADL dependency in *M*_*RE*_^*d*^_*0*_ models was on average 0.94 (0.93–0.95), accuracy 0.90 (0.89–0.91), precision 0.91 (0.89–0.92), and recall 0.90 (0.84–0.95). When predicting functional status up to one year ahead, $\tau \in \left\{ {90,180,365} \right\}$, the *M*_*RE*_^*d*^_*τ*_ models’ accuracy drops to AUC 0.77 (0.73–0.79), accuracy 0.73 (0.69–0.80), precision 0.74 (0.66–0.81), and recall 0.69 (0.34–0.96). When the previous functional status is unknown (i.e., initial evaluation), the performance of the current assessment models *M*_*E*_^*d*^_*0*_ decreased by about 16% (*p* < 0.01) in terms of AUC. On average, the obtained results for these models are AUC 0.79, accuracy 0.74, precision 0.74, and recall 0.80. A complete set of results for individual models is available in Additional file 2.

**Table 4:** Average ± standard deviation of accuracy, AUC, precision and recall of models in predicting functional status

	Re-evaluation models (*M*_*RE*_^*d*^_*τ*_*)*				Evaluation models (*M*_*E*_^*d*^_*τ*_*)*			
Prediction time τ	Accuracy	AUC	Precision	Recall	Accuracy	AUC	Precision	Recall
Current	.900 ± .007	.947 ± .006	.910 ± .011	.907 ± .041	.743 ± .029	.795 ± .010	.743 ± .046	.800 ± .128
3 Months	.815 ± .020	.876 ± .011	.849 ± .019	.816 ± .094	.727 ± .037	.761 ± .006	.734 ± .049	.783 ± .161
6 Months	.759 ± .029	.808 ± .014	.784 ± .029	.737 ± .165	.720 ± .038	.746 ± .009	.721 ± .045	.729 ± .238
12 Months	.737 ± .035	.772 ± .022	.742 ± .049	.699 ± .226	.716 ± .039	.725 ± .016	.696 ± .073	.701 ± 264

### Top predictors

Further analysis also identified the top predictors used in the assessment and prediction of ADLs. Average Gini Index [38] produced by random forest was used to measure the quality of predictors. Gini index is a data impurity measure used in the presented work by random forest as an internal measure of attribute quality when constructing individual decision trees. It should not be interpreted as a strength or effect of the variable on the predicted output, but rather to understand the relative importance of attributes. In general, random forests can use many other attribute quality measures, but model tuning indicated that Gini index performs the best in CBIT. Top predictors along with their reported importance (average Gini index over all trees in forest and over all models) are presented in Table 5. Note that all *ccs*_*i*_^*min*^ and *ccs*_*i*_^*max*^ codes were included in full models. A longer list of diagnosis codes and previous evaluations are available in Additional file 3. Not surprisingly, the most predictive attributes in *M*_*RE*_^*d*^_*τ*_ models were past functional status, being responsible for AUC of 0.93. Other most predictive attributes were the time since the most recent diagnosis of delirium, dementia, and amnestic and other cognitive disorders (CCS 653) and patient age. These were followed by encoded time of diagnoses/administrative codes for: the urinary tract infections (CCS 159); chronic ulcer of skin (CCS 199); other connective tissue disease (CCS 211); paralysis (CCS 82); administrative/social admission (CCS 255); alcohol-related disorders (CCS 660); aspiration pneumonitis; food/vomitus (CCS 129); and schizophrenia and other psychotic disorders (CCS 659). For most of the diagnoses listed above, it is important when (number of days) a patient was diagnosed with that condition most recently. For ulcers and aspiration pneumonitis; food/vomitus, the first diagnosis is important. In addition, the table has marked potentially reversible conditions (R), as judged by clinicians, which can be influenced in the care provided to the patients and affect the outcome.

**Table 5:** Top ranked predictors of functional status

Rank	Attributes	Min/Max	Description	R	GINI RE-EVAL	GINI EVAL
1	ccs653	Min	Delirium, dementia, and amnestic and other cognitive disorders		0.0216	0.0310
2	Age		Age at the time of prediction		0.0133	0.0335
3	ccs159	Min	Urinary tract infections	X	0.0128	0.0217
4	ccs199	Max	Chronic ulcer of skin		0.0071	0.0121
5	ccs211	Min	Other connective tissue disease		0.0065	0.0091
6	ccs82	Min	Paralysis	X	0.0062	0.0110
7	ccs255	Min	Administrative/social admission	X	0.0061	0.0107
8	ccs660	Min	Alcohol-related disorders	X	0.0058	0.0110
9	ccs129	Max	Aspiration pneumonitis; food/vomitus		0.0055	0.0072
10	cs659	Min	Schizophrenia and other psychotic disorders		0.0055	0.0089
	…					
337	W		Race White		0.0006	0.0012
341	UR		Unknown Race		0.0006	0.0011
365	B		Race Black		0.0004	0.0009
434	Gender		Gender		0.0002	0.0004
445	A		Race Asian		0.0002	0.0003

“GINI RE-EVAL” indicates score of a variable in Re-Evaluation models (*M*_*RE*_^*d*^_*τ*_*)*. “GINI EVAL” indicates score of a variable in Evaluation models (*M*_*E*_^*d*^_*τ*_*)*. R are potentially reversible or red flag that this person is at risk and needs restorative therapy; Race and Gender variables are included at the bottom of the table for comparison but have very low impact on prediction

### Simplified models

**Fig. 3:**
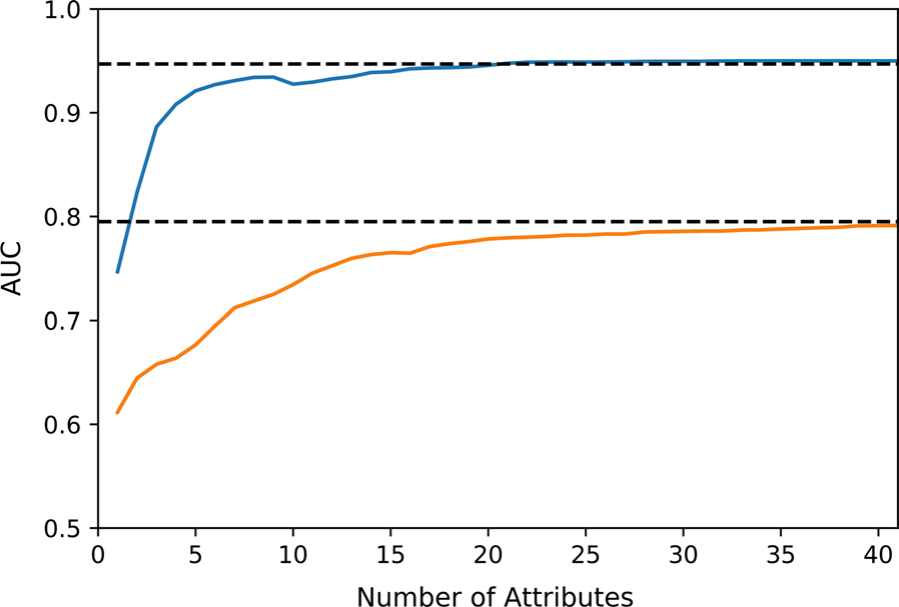
Average AUC for the current assessment based on the number of attributes used. The blue line refers to *MS*_*RE*_^*d*^_*τ*_ models and the orange line refers to *MS*_*E*_^*d*^_*τ*_ models

Further, simplified models (called *MS*_*RE*_^*d*^_*τ*_ and *MS*_*E*_^*d*^_*τ*_*)* that include only selected top-ranking patient characteristics were developed. Average GINI score was used to rank attributes. As depicted in Fig. 3, adding more characteristics beyond the most predictive 41 attributes did not significantly improve the accuracy (*p* < 0.05) of the models in assessing the current functional status (τ = 0) as compared to full model. The curves were also similar for predicting up to 12 months ahead, $\tau \in \left\{ {90,180,365} \right\}$. When using 25 top patient characteristics, models that included previous evaluations (*MS*_*RE*_^*d*^_*τ*_) reached an average AUC of 0.94, accuracy 0.90, precision 0.91, and recall 0.90. Furthermore, the performance of the simplified models with 41 patient characteristics and without previous evaluations (*MS*_*E*_^*d*^_*τ*_) raised to average AUC of 0.79, accuracy 0.74, precision 0.74, and recall 0.78. Note that top predictors for each ADL are different. In the *MS*_*RE*_^*d*^_*τ*_ and *MS*_*E*_^*d*^_*τ*_ models, top ranking attributes were included across all models to minimize information needed by CBIT for all ADLs, even though this set of attributes may not be optimal for individual models.

### Temporal coding

One important advancement of the presented CBIT is the way it captures time in encoding diagnoses as previously shown in Eqs. (1) and (2) and illustrated in Table 3. The proposed method of constructing attributes for diagnoses was investigated to determine how it would be different from binary attributes (1 when a diagnosis is present in a given patient’s record and 0 otherwise) when used in CBIT. All constructed *M*_*RE*_^*d*^_*τ*_*, M*_*E*_^*d*^_*τ*_, *MS*_*RE*_^*d*^_*τ*_*,* and *MS*_*E*_^*d*^_*τ*_ models were compared in terms of AUC at different time points up to one year ahead. In one experiment, random forest was compared with other algorithms including logistic regression, decision tree, and naïve Bayes.

As mentioned earlier, when temporal attributes are used, one needs to assign special values to diagnoses that are not present in data. Therefore, ± 999,999 (6_9) was compared with ± 9999 (4_9), and ± 99,999 (5_9) coding across all models (here X_9 indicates 10^X^-1). Temporal coding (6_9) was also compared with binary coding to determine any significant difference. Two-tailed t-test was used to assess all comparisons (*p* < 0.05).

As summarized in Table 6, both random forest and logistic regression show a significant difference in AUC when temporal information is applied (*p* < 0.05). The results indicated that random forest with the temporal coding performs significantly better than binary coding, while for the logistic regression the relationship is opposite (the binary coding is better). However, logistic regression with binary coding is still doing worse than random forest. Decision trees and naïve Bayes results were also included in the table, but the performance was typically inferior. It was observed that random forest, decision tree and naïve Bayes are not affected by how the special values were assigned, while the performance of logistic regression is affected by the coding. The rationale for this result is that for symbolic methods it is irrelevant how not-present values are coded as long as the value is distinct, while parametric models need to find a coefficient for each diagnosis code, which is affected by the coding.

**Table 6:** Comparison of temporal and binary diagnosis coding as part of CBIT construction and evaluation

AUC	Current assessment				3 month prediction				6 month prediction				12 month prediction			
	RF	LR	DT	NB	RF	LR	DT	NB	RF	LR	DT	NB	RF	LR	DT	NB
***M***_***RE***_^***d***^_***τ***_																
Temporal 4_9	0.95^*^	0.85^*+^	0.92^+^	0.87^*+^	0.88	0.79^*+^	0.83^+^	0.83^+^	0.81	0.77^*+^	0.74^+^	0.78^+^	0.77	0.74^*+^	0.70^+^	0.74^+^
Temporal 5_9	0.95	0.78^*+^	0.92^+^	0.89^+^	0.88	0.76^*+^	0.83^+^	0.83^+^	0.81	0.74^+^	0.74^*+^	0.78^+^	0.77	0.71^*+^	0.70^+^	0.74^+^
Temporal 6_9	0.95	0.78^+^	0.92^+^	0.90^+^	0.88	0.75^+^	0.83^+^	0.83^+^	0.81	0.74^+^	0.74^+^	0.78^+^	0.77	0.72^+^	0.70^+^	0.74^+^
Binary	0.94^*^	0.94^*^	0.91^*+^	0.87^*+^	0.87^*^	0.87^*+^	0.82^*+^	0.80^+^	0.81	0.81^*+^	0.74^+^	0.77^*+^	0.77	0.77^*+^	0.70^+^	0.74^*+^
***MS***_***RE***_^***d***^_***τ***_																
Temporal 4_9	0.95	0.94^*+^	0.92^+^	0.89^+^	0.88	0.88^*+^	0.83^+^	0.82^+^	0.81^*^	0.81^*+^	0.74^+^	0.76^+^	0.77	0.77^*^	0.70^+^	0.72^+^
Temporal 5_9	0.95	0.93^*+^	0.92^+^	0.89^+^	0.88	0.84^*+^	0.82^+^	0.82^+^	0.81^*^	0.79^*+^	0.74^+^	0.76^+^	0.77	0.75^*+^	0.70^+^	0.72^+^
Temporal 6_9	0.95	0.76^+^	0.92^+^	0.90^+^	0.88	0.72^+^	0.83^+^	0.82^+^	0.81	0.71^+^	0.74^+^	0.76^+^	0.77	0.69^+^	0.70^+^	0.72^+^
Binary	0.94^*^	0.94^*+^	0.90^*+^	0.90^+^	0.88^*^	0.87^*+^	0.81^*+^	0.83^+^	0.81^*^	0.81^*+^	0.74^*+^	0.78^*+^	0.77	0.77^*^	0.69^+^	0.74^*+^
***M***_***E***_^***d***^_***τ***_																
Temporal 4_9	0.79	0.79^*+^	0.72^*+^	0.73^+^	0.76	0.76^*^	0.68^+^	0.68^+^	0.75^*^	0.75^*^	0.66^+^	0.71^+^	0.73	0.72^*^	0.64^+^	0.69^+^
Temporal 5_9	0.79	0.78^*+^	0.71^+^	0.73^+^	0.76	0.75^+^	0.68^+^	0.68^+^	0.75	0.74^*+^	0.66^+^	0.71^+^	0.73	0.71^*+^	0.64^+^	0.69^+^
Temporal 6_9	0.79	0.78^*^	0.72^+^	0.73^+^	0.76	0.75^+^	0.68^+^	0.68^+^	0.75	0.74	0.66^+^	0.71^+^	0.73	0.72^+^	0.64^+^	0.69^+^
Binary	0.78^*^	0.78^*^	0.70^*+^	0.73^+^	0.76	0.76^*^	0.67^*+^	0.70^*+^	0.75	0.75^*^	0.66^+^	0.71^*+^	0.72^*^	0.73^*+^	0.64^+^	0.69^*+^
***MS***_***E***_^***d***^_***τ***_																
Temporal 4_9	0.79	0.77^*+^	0.71^+^	0.64^+^	0.76	0.75^*^_+_	0.68^+^	0.63^+^	0.74	0.73^*+^	0.66^+^	0.60^+^	0.72	0.72^*^	0.63^+^	0.58^+^
Temporal 5_9	0.79	0.76^*+^	0.71^+^	0.64^+^	0.76	0.73^*+^	0.68^+^	0.63^+^	0.74	0.72^*+^	0.66^+^	0.60^+^	0.72	0.71^*+^	0.63^+^	0.58^+^
Temporal 6_9	0.79	0.75^+^	0.71^+^	0.64^+^	0.76	0.72^+^	0.68^+^	0.63^+^	0.74	0.71^+^	0.66^+^	0.60^+^	0.72	0.69^+^	0.63^+^	0.58^+^
Binary	0.76^*^	0.77^*+^	0.68^*+^	0.74^*+^	0.74^*^	0.74^*^	0.65^*+^	0.71^*+^	0.73^*^	0.73^*^	0.64^*+^	0.71^*+^	0.71^*^	0.72^*+^	0.63^+^	0.69^*+^

The results are presented in terms of AUC for the current assessment and prediction up to 12 months ahead. Full models that include 578 attributes and simplified models with 50 attributes are shown. 4_9, 5_9, and 6_9 indicate the encoding of diagnoses not present in patient’s history for ± 9999, ± 99,999, and ± 999,999, respectively. *Indicates significance (*p* < 0.05) of coding systems compared to “6_9” and + indicates significance (*p* < 0.05) of different algorithms compared to random forest

### Calibration

**Fig. 4:**
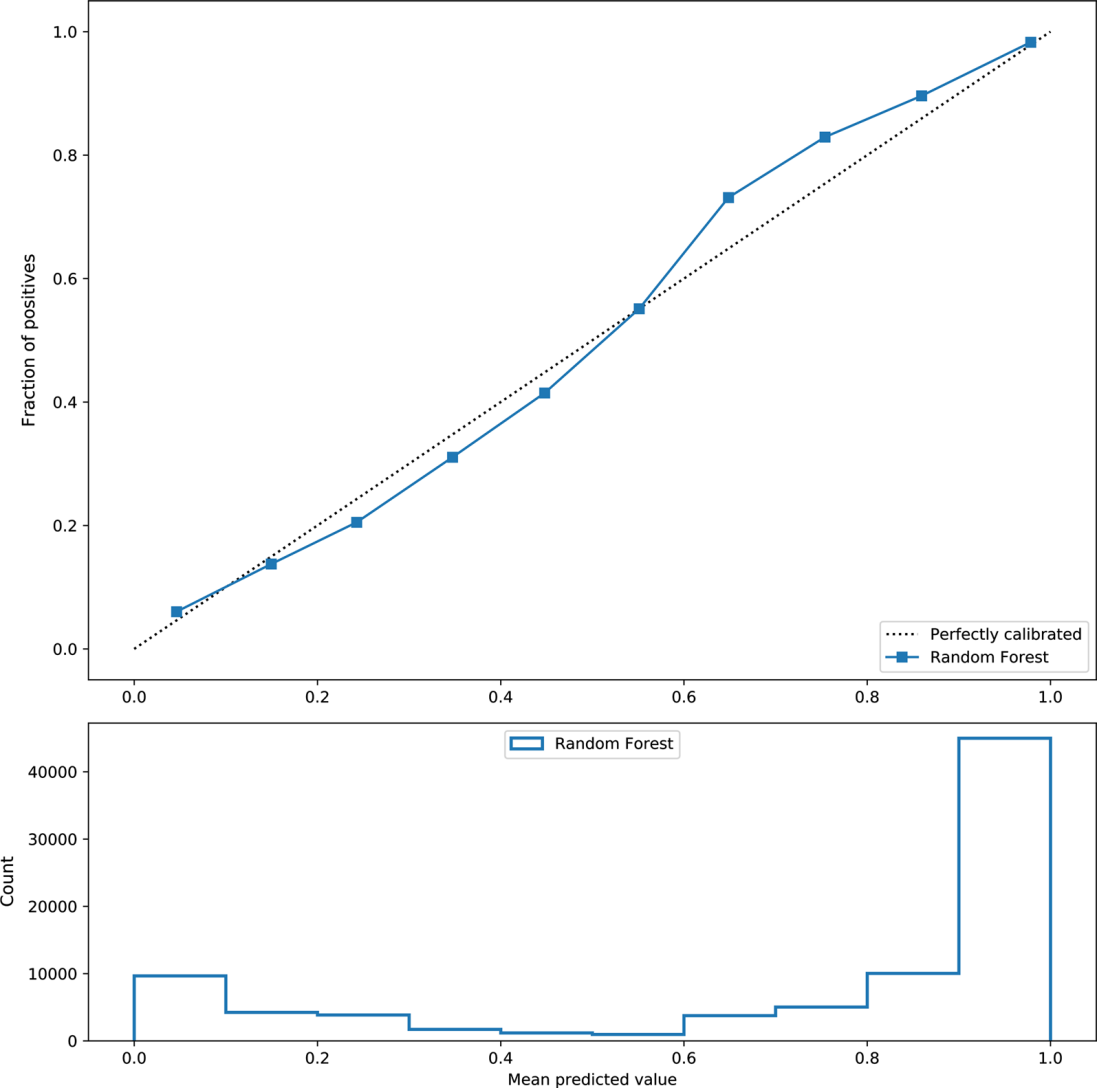
Example of the calibration curve for model that predicts bathing at current time point. The shape of the curve indicates that the model is well-calibrated. Similar curves were created for all models used in CBIT

Calibration allows for the probability interpretation of the output scores from the models, further allowing for frequency interpretation of the results. Thus, all models were calibrated using fivefold cross-validated isotonic regression. This approach fits a secondary model on top of the created random forest models and attempts to adjust returned scores make them closer to probabilities. The results showed that the models were well-calibrated with mean squared error of about 3%. Figure 4 shows an example of the calibration curve for the model that assesses bathing at the current time point, *MS*_*RE*_^*bathing*^_*0*_. Similar curves were also developed for all 144 models and are available in supplemental materials (See Additional files 8–11).

## Discussion

### Methods

It was shown that it is possible to assess and predict functional status using machine learning methods. Moreover, it was shown that the inclusion of time between diagnosis and time of prediction is important in constructing attributes in the data. While further work is needed to validate the new way of constructing attributes representing diagnoses and study its limitations, counting days from the first and last known occurrence of a diagnosis code works for the problem at hand.

Machine learning methods are gaining popularity in medical and health applications, yet there is no consensus on what validation is needed for their use in clinical settings. There is also no agreement about what information is needed to allow for full reproducibility of ML results, or even what reproducibility in this context means [43]. Models created for CBIT were evaluated using standard measures in ML model testing (cross-validation, independent test set, etc.), and investigated in terms of their calibration and learning curves. There is a need for further validation of the models and their impact on patient care. Such validation focuses on detailed model analysis in terms of accuracy, transparency and the ability to provide explanations, and eventually trust and acceptability by the medical community. A randomized trial to assess outcomes of the model use may be required for full acceptance in clinical settings.

In addition, there is an ongoing discussion about the overall validity of applying machine learning methods to the prediction of patient outcomes, and potential bias of the models based on gender, race and socioeconomic status. One needs to clearly understand data limitations and definitions of the prediction problem to understand the drawbacks of the method. Supervised machine learning methods, by definition, learn what they are asked to learn, and may (typically do) propagate biases from training data. Biases in machine learning-based models are typically not caused by machine learning, but by underlying process used to create training data. The key is in the definition and construction of the output attributes of the model and their proper interpretation. One needs to answer a question if models predict events in the real world, or data artifacts that somehow approximate that reality. Similarly, CBIT is intended to mimic the tasks of nurses performing evaluations of ADLs as part of the MDS. Therefore, any biases, inaccuracies, or subjectivity in this process may also be repeated by the CBIT models. However, in CBIT, as shown in Table 5, race and gender had only a negligible impact on predictions and were completely dropped from simplified models, which suggests diminishing the potential racial and gender bias in the models. A different set of methods, typically used in health services research, is needed to understand the existence of potential bias in constructed models.

The probabilistic interpretation of the prediction results used in the presented work seems to be reasonable. Conceptually, the future can never be predicted with the probability of one (even though for some cases the models may be certain of the future and output 1.0). Instead, the values represent how likely an event (here functional independence) will occur according to the models. Such interpretation has several advantages. It allows end users to interpret the chances of an event happening, and in turn, describes when models are uncertain about the predicted outcomes. In applications such as the presented CBIT, providing probability as a form of explanation makes the predictions more transparent. Knowing how likely an outcome is going to happen can helps clinicians, patients and their families make informed decisions related to planning care. This is in contrast with systems in which ML-based models trigger certain event such as alerts within EHR systems. Such triggered events are binary in nature (alert or no alert), thus, the final assigned class is most important. The probability results also explain why model accuracy is not 100% when executed on the test data (examples with predicted probability not equal to one, are ambiguous by the definition of probability). The latter is the most evident when analyzing calibration curves, such as one presented in Fig. 4. The disadvantage of using the probabilities is that they may be misinterpreted as severity of disability. When presenting results, one needs to specify that the number represent how likely a patient is independent, and not the level of dependency.

The created models in this manuscript are based on ICD-9 diagnosis codes, which were mapped to CCS codes. One advantage of this approach is that since all new data are coded with ICD-10 codes, they could easily be mapped to CCS codes making the models applicable to data with the newer coding system. Another important issue is that the diagnosis codes in both EHR and claims data are subject to under- and over-coding, thus affecting the potential reliability of the models. However, it is important to note that our modeling efforts were not intended to understand the effects of diagnoses on ADLs, but rather their use in making prediction. In addition, as long as diagnoses are systematically over/under-coded, they should not affect performance of the models. Despite these limitations, results indicated that our data were appropriate for this purpose.

The evaluations presented in this paper are only summaries and examples of detailed results. A detailed examination of all 72 *M*^*d*^_*τ*_ models that are part of CBIT and 72 *MS*^*d*^_*τ*_ models that are part of the limited CBIT was performed, and the results are available in the supplemental materials and through the online calculator [44]. All developed models and source codes are available for everyone who wishes to conduct their testing on independent data from other institutions, i.e. to test cross-institution generalizability.

### Clinical and administrative use

Very little evidence exists to address whether measuring functional status can change the quality of life, but our research shows that prior knowledge about functional disability is a key indicator of future functional status. Notably, past research has provided evidence that improvements in functional status are possible over time through therapy [45] by improving, slowing decline, and/or maintaining functional status. The presented CBIT tool which predicts improvement or decline could be used by health professionals as means of identifying patient characteristics that are modifiable and plan care accordingly. It can serve as a basis for an informed discussion between clinicians, patients and caregivers. In addition, these measures could potentially serve as a patient-centered measure for examining the value of the services provided.

### Graphical presentation of results and web calculator

**Fig. 5:**
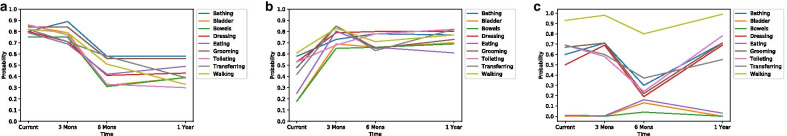
Predicted probability visualization of functional independence for three patients up to one year ahead

A graphical representation of the assessment and prediction of functional status can be used by healthcare professionals and caregivers for decision making regarding the patients’ care. Our full models can be integrated as decision support tools within EHR systems or linked to claims data, while the simplified models can operate standalone as an interactive online tool. For example, Fig. 5 illustrates CBIT-predicted outcomes for three fictitious patients similar to what was shown in Fig. 1. Values indicate the probability of functional independence for each ADL up to one year after the prediction time. The higher the value is, the higher the chance that the patient is functionally independent. One can observe significant differences between the functional dependency trajectories for these patients. Patient (a) is currently likely to be independent but expected to decline within 6 months as the probability of independence decreases. Patient (b) is currently likely to be dependent in most ADLs (probability of independence ranging from 0.2 to 0.6) but predicted to recover in the next 3 months and stay at this level afterward. Patient (c) is independent and predicted to remain independent in terms of walking and is almost certainly disabled in terms of bladder, bowels and eating. The patient is likely to have a temporary decline in terms of other ADLs. Construction of each of the plots requires execution of 36 random forest models (9 ADLs, 4 time points).

An experimental version of the online calculator that takes patient characteristics and outputs plots is available at https://hi.gmu.edu/cbit [44]. It is accessible through a web form or an application programming interface (API). The web calculator is implemented in Python 3 and uses Flask as a web application framework, with Pandas and Scikit-learn libraries performing data analysis. To ensure the performance of the web calculator, all of the models are loaded on the startup and reside in RAM. Additional changes have been made to the calculator to improve clinical use. For example, for the ease of use, the numbers of days associated with diagnoses were discretized to allow users to select them from drop-down menus. Numbers closer to zero are discretized with higher precision than larger numbers, which further improves understandability. Users can enter patient information and are provided with results similar to those shown in Fig. 5 along with a data table containing the values of predicted probabilities. An explanation module that provides human-oriented interpretation of the results as well as the reasons for predictions is in development.

## Conclusion

This study found that functional status can be assessed and predicted with high accuracy when prior functional status in medical history is available, but also without requiring previous in-person functional assessment. It exemplifies an opportunity of applying machine learning to large data to produce meaningful results. It was hypothesized that a parsimonious model could be developed with variables available in EHRs or claims data and assumed that this model would retain predictive accuracy for up to a year ahead. Our experimental results confirmed this hypothesis. The constructed tool is intended to be used in both clinical and administrative settings and has implications for caregivers, clinicians, and policy makers. Assessment and prediction of functional status may also lead to better care planning for nursing home residents as well as the elderly residing in their own homes. Automated large-scale assessment and prediction of functional status can be used to compare care settings and as a benchmark for provider outcomes.

The constructed full model requires a large number of predictors, which makes it impossible to manually enter values. Hence, the full version of CBIT would need to be integrated with an EHR or claims management system to be part of the clinical decision support. Such integration can be achieved using HL7s FHIR interface. The simplified version of the CBIT that uses 50 predictors is available within a web calculator. Beyond the use of EHR data, the constructed CBIT could be enhanced by sensor data allowing for continuous patient monitoring and be integrated with the presented approach. Such data can aid assessment, particularly for ADLs that measure patient movement [46–48].

The presented work has a number of limitations. The tool is not applicable in settings in which longitudinal patient records are not available. Only large health systems with long-established electronic medical records have sufficient longitudinal data to apply models that use temporal diagnosis information. Additionally, the models were developed using data from the US Department of Veterans Affairs (VA), which does not reflect the general population of nursing home residents outside of the VA system. The performance of these models on other datasets, including Medicare claims data are being investigated. It is unclear how the models will perform on a very different population and if the existing CBIT models can be adapted. Finally, random forests are known to be “black box” models that work well but are not well understood by end users. Even though their explanation is easier than other types of models such as neural networks, they are significantly more difficult than linear models, decision trees or decision rules. Instead of trying to explain the entire model (as part of the online calculator), there is an ongoing effort in designing an explanation module that provides users with “reasons” for making specific predictions in one individual case (prediction explanation). The reasons consist of a list of patient characteristics that are the strongest predictors (both confirming and disconfirming) for that individual case. Despite these limitations, CBIT can be used to support clinicians and administrators in decision making. Our novel data coding method, applying machine learning to unique health data, comprehensive model testing, and transparency of the work contribute to the state-of-the-art in ML-based decision support.

## Supplementary Information

**Additional file 1.** Barthel Index categories of functional abilities along with assigned scores. Reproduced from: http://www.strokecenter.org/wp-content/uploads/2011/08/bartel.pdf.

**Additional file 2.** Detailed performance of the models. Detailed testing results of 72 CBIT models, reported in terms of AUC, accuracy, precision, recall and F1-score.

**Additional file 3.** Top ranked predictors of functional status. The table includes top 50 attributes across Re-Evaluation and Evaluation models. Previous evaluations results associated with the Re-Evaluation models (MREdτ) were included at the beginning of the table. Gender and race along with their ranking were also added at the bottom of the table for comparison.

**Additional file 4.** Learning_Curves_Full_Evaluation_Models. The file includes 36 learning curves for Full Evaluation Models in CBIT.

**Additional file 5.** Learning_Curves_Full_Re-Evaluation_Models. The file includes 36 learning curves for Full Re-Evaluation Models in CBIT.

**Additional file 6.** Learning_Curves_Simplified_Evaluation_Models. The file includes 36 learning curves for Simplified Evaluation Models in CBIT.

**Additional file 7.** Learning_Curves_Simplified_Re-Evaluation_Models. The file includes 36 learning curves for Simplified Re-Evaluation Models in CBIT.

**Additional file 8.** Calibration_Plots_Full_Evaluation_Models. The file includes 36 calibration plots for Full Evaluation Models in CBIT.

**Additional file 9.** Calibration_Plots_Full_Re-Evaluation_Models. The file includes 36 calibration plots for Full Re-Evaluation Models in CBIT.

**Additional file 10.** Calibration_Plots_Simplified_Evaluation_Models. The file includes 36 calibration plots for Simplified Evaluation Models in CBIT.

**Additional file 11.** Calibration_Plots_Simplified_Re-Evaluation_Models. The file includes 36 calibration plots for Simplified Re-Evaluation Models in CBIT.

## Data Availability

The data used to construct presented CBIT models are individual level and cannot be shared. Access to the original data may be requested through the US Department of Veterans Affairs. All constructed models, source code, and detailed testing results are freely available at https://hi.gmu.edu/cbit.

## Abbreviations

ADL: Activity of Daily Living
CBIT: Computational Barthel Index Tool
VA: Department of Veterans Affairs
MDS: Minimum Data Set
EHR: Electronic Health Record
ML: Machine learning
ICD-9: International Classification of the Diseases, ninth edition
CCS: Clinical Classification Software
AHRQ: Agency of Health Research and Quality Control
AUC: Area under the curve
RF: Random forest
LG: Logistic Regression
DT: Decision tree
NB: Naïve Bayes
ICD-10: International Classification of the Diseases, tenth edition
API: Application programming interface
RAM: Random access memory
