# Supplementary material for: Computational Barthel Index: an automated tool for assessing and predicting activities of daily living among nursing home patients
Source: BMC Med Inform Decis Mak. 2021 Jan 9;21:17. doi: 10.1186/s12911-020-01368-8 (PMC7796534; doi:10.1186/s12911-020-01368-8)
Supplement: Supplementary file 1 — Additional file 1. Barthel Index categories of functional abilities along with assigned scores. Reproduced from: http://www.strokecenter.org/wp-content/uploads/2011/08/bartel.pdf. [file 12911_2020_1368_MOESM1_ESM.pdf]

Additional file 1 for Wojtusiak et al., Computational Barthel Index: An Automated Tool for Assessing and Predicting Activities of Daily Living Among Nursing Home Patients

Barthel Index categories of functional abilities along with assigned scores. Reproduced from: http://www.strokecenter.org/wp-content/uploads/2011/08/barthel.pdf

Item	Levels
Feeding	0 = unable, 5 = Needs help cutting; spreading butter, etc., or requires modified diet; 10 = independent
Bathing	0=dependent; 5=independent (or in shower)
Grooming	0 = needs to help with personal care; 5 = independent face/hair/teeth/shaving (implements provided)
Dressing	0 = dependent; 5 = needs help but can do about half unaided; 10 = independent (including buttons, zips, laces, etc.)
Bowels	0 = incontinent (or needs to be given enemas); 5 = occasional accident; 10 = continent
Bladder	0 = incontinent, or catheterized and unable to manage alone; 5 = occasional accident; 10 = continent
Toilet Use	0 = dependent; 5 = needs some help, but can do something alone; 10 = independent (on and off, dressing, wiping)
Transfers (Bed to chair and back)	0 = unable, no sitting balance; 5 = major help (one or two people, physical), can sit; 10 = minor help (verbal or physical); 15 = independent
Mobility (On Level Surfaces)	0 = immobile or < 50 yards; 5 = wheelchair independent, including corners, > 50 yards; 10 = walks with help of one person (verbal or physical) > 50 yards; 15 = independent (but may use any aid; for example, stick) > 50 yards
Stairs	0 = unable; 5 = needs help (verbal, physical, carrying aid); 10 = independent

Use of stairs is not included in the presented CBIT method.
