# Supplementary material for: Computational Barthel Index: an automated tool for assessing and predicting activities of daily living among nursing home patients
Source: BMC Med Inform Decis Mak. 2021 Jan 9;21:17. doi: 10.1186/s12911-020-01368-8 (PMC7796534; doi:10.1186/s12911-020-01368-8)
Supplement: Supplementary file 3 — Additional file 3. Top ranked predictors of functional status. The table includes top 50 attributes across Re-Evaluation and Evaluation models. Previous evaluations results associated with the Re-Evaluation models (MREdτ) were included at the beginning of the table. Gender and race along with their ranking were also added at the bottom of the table for comparison. [file 12911_2020_1368_MOESM3_ESM.pdf]

# Additional file 3 for Wojtusiak et al., Computational Barthel Index: An Automated Tool for Assessing and Predicting Activities of Daily Living Among Nursing Home Patients

**Top ranked predictors of functional status.**

| Rank | Attributes | Min/Max | Description | R | GINI RE-EVAL | GINI EVAL |
|---|---|---|---|---|---|---|
| 1 | Eating | | Previous known score for eating | | 0.2522 | ----- |
| 2 | Bladder | | Previous known score for bladder incontinence | | 0.2391 | ----- |
| 3 | Walking | | Previous known score for walking | | 0.2106 | ----- |
| 4 | Bathing | | Previous known score for bathing | | 0.1911 | ----- |
| 5 | Transferring | | Previous known score for transferring | | 0.1907 | ----- |
| 6 | Bowels | | Previous known score for bowel incontinence | | 0.1872 | ----- |
| 7 | Dressing | | Previous known score for dressing | | 0.1724 | ----- |
| 8 | Grooming | | Previous known score for grooming | | 0.1694 | ----- |
| 9 | Toileting | | Previous known score for toileting | | 0.1694 | ----- |
| 10 | ccs653 | Min | Delirium, dementia, and amnestic and other cognitive disorders | | 0.0216 | 0.0310 |
| 11 | Age | | Age at the time of prediction | | 0.0133 | 0.0335 |
| 12 | ccs159 | Min | Urinary tract infections | X | 0.0128 | 0.0217 |
| 13 | ccs199 | Max | Chronic ulcer of skin | | 0.0071 | 0.0121 |
| 14 | ccs211 | Min | Other connective tissue disease | | 0.0065 | 0.0091 |
| 15 | ccs82 | Min | Paralysis | X | 0.0062 | 0.0110 |
| 16 | ccs255 | Min | Administrative/social admission | X | 0.0061 | 0.0107 |
| 17 | ccs660 | Min | Alcohol-related disorders | X | 0.0058 | 0.0110 |
| 18 | ccs129 | Max | Aspiration pneumonitis; food/vomitus | | 0.0055 | 0.0072 |
| 19 | ccs659 | Min | Schizophrenia and other psychotic disorders | | 0.0055 | 0.0089 |
| 20 | ccs155 | Min | Other gastrointestinal disorders | X | 0.0053 | 0.0113 |
| 21 | ccs257 | Min | Other aftercare | | 0.0053 | 0.0077 |
| 22 | ccs98 | Min | Essential hypertension | | 0.0052 | 0.0089 |
| 23 | ccs95 | Min | Other nervous system disorders | | 0.0050 | 0.0066 |
| 24 | ccs663 | Max | Screening and history of mental health and substance abuse codes | | 0.0049 | 0.0078 |
| 25 | ccs162 | Min | Other diseases of bladder and urethra | | 0.0045 | 0.0040 |
| 26 | ccs113 | Min | Late effects of cerebrovascular disease | X | 0.0045 | 0.0082 |
| 27 | ccs259 | Min | Residual codes; unclassified | | 0.0044 | 0.0067 |
| 28 | ccs254 | Min | Rehabilitation care; fitting of prostheses; and adjustment of devices | X | 0.0043 | 0.0068 |
| 29 | ccs256 | Min | Medical examination/evaluation | | 0.004 | 0.0074 |
| 30 | ccs258 | Min | Other screening for suspected conditions (not mental disorders or infectious disease) | | 0.0040 | 0.0061 |

| | | | | | | |
|---|---|---|---|---|---|---|
| 31 | ccs163 | Min | Genitourinary symptoms and ill-defined conditions | X | 0.0037 | 0.0059 |
| 32 | ccs59 | Min | Deficiency and other anemia | | 0.0037 | 0.0047 |
| 33 | ccs53 | Min | Disorders of lipid metabolism | | 0.0037 | 0.0076 |
| 34 | ccs136 | Max | Disorders of teeth and jaw | | 0.0036 | 0.0066 |
| 35 | ccs204 | Min | Other non-traumatic joint disorders | | 0.0036 | 0.0071 |
| 36 | ccs661 | Min | Substance-related disorders | X | 0.0036 | 0.0077 |
| 37 | ccs89 | Min | Blindness and vision defects | X | 0.0035 | 0.0070 |
| 38 | ccs197 | Min | Skin and subcutaneous tissue infections | X | 0.0034 | 0.0047 |
| 39 | ccs114 | Min | Peripheral and visceral atherosclerosis | | 0.0034 | 0.0038 |
| 40 | ccs3 | Max | Bacterial infection; unspecified site | | 0.0032 | 0.0045 |
| 41 | ccs10 | Min | Immunizations and screening for infectious disease | | 0.0031 | 0.0052 |
| 42 | ccs109 | Min | Acute cerebrovascular disease | X | 0.0030 | 0.0067 |
| 43 | ccs238 | Min | Complications of surgical procedures or medical care | | 0.0029 | 0.0041 |
| 44 | ccs127 | Min | Chronic obstructive pulmonary disease and bronchiectasis | X | 0.0028 | 0.0052 |
| 45 | ccs86 | Min | Cataract | | 0.0026 | 0.0058 |
| 46 | ccs205 | Min | Spondylosis; intervertebral disc disorders; other back problems | X | 0.0027 | 0.0058 |
| 47 | ccs200 | Min | Other skin disorders | X | 0.0025 | 0.0055 |
| 48 | ccs45 | Min | Maintenance chemotherapy; radiotherapy | | 0.0016 | 0.0053 |
| 49 | ccs657 | Max | Mood disorders | | 0.0026 | 0.0048 |
| 50 | ccs111 | Min | Other and ill-defined cerebrovascular disease | X | 0.0025 | 0.0047 |
| | … | | | | | |
| 337 | W | | Race White | | 0.0006 | 0.0012 |
| 341 | UR | | Unknown Race | | 0.0006 | 0.0011 |
| 365 | B | | Race Black | | 0.0004 | 0.0009 |
| 434 | Gender | | Gender | | 0.0002 | 0.0004 |
| 445 | A | | Race Asian | | 0.0002 | 0.0003 |

The table includes top 50 attributes across Re-Evaluation and Evaluation models. Previous evaluations results associated with the Re-Evaluation models ($M_{RE}{}^{d}{}_{\tau}$) were included at the beginning of the table. Gender and race along with their ranking were also added at the bottom of the table for comparison.
