# Supplementary figures and images for: Computational Barthel Index: an automated tool for assessing and predicting activities of daily living among nursing home patients
Source: BMC Med Inform Decis Mak. 2021 Jan 9;21:17. doi: 10.1186/s12911-020-01368-8 (PMC7796534; doi:10.1186/s12911-020-01368-8)

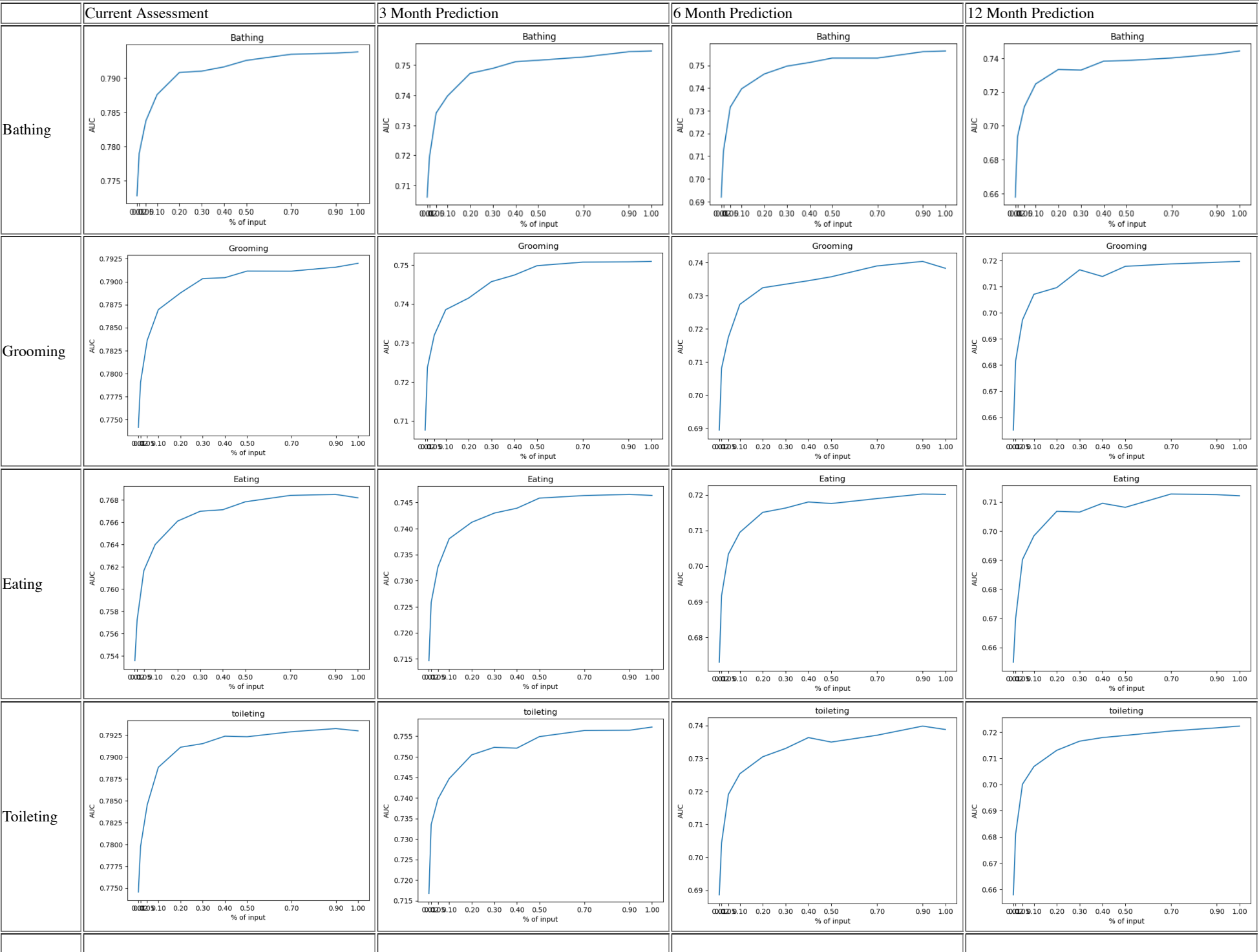

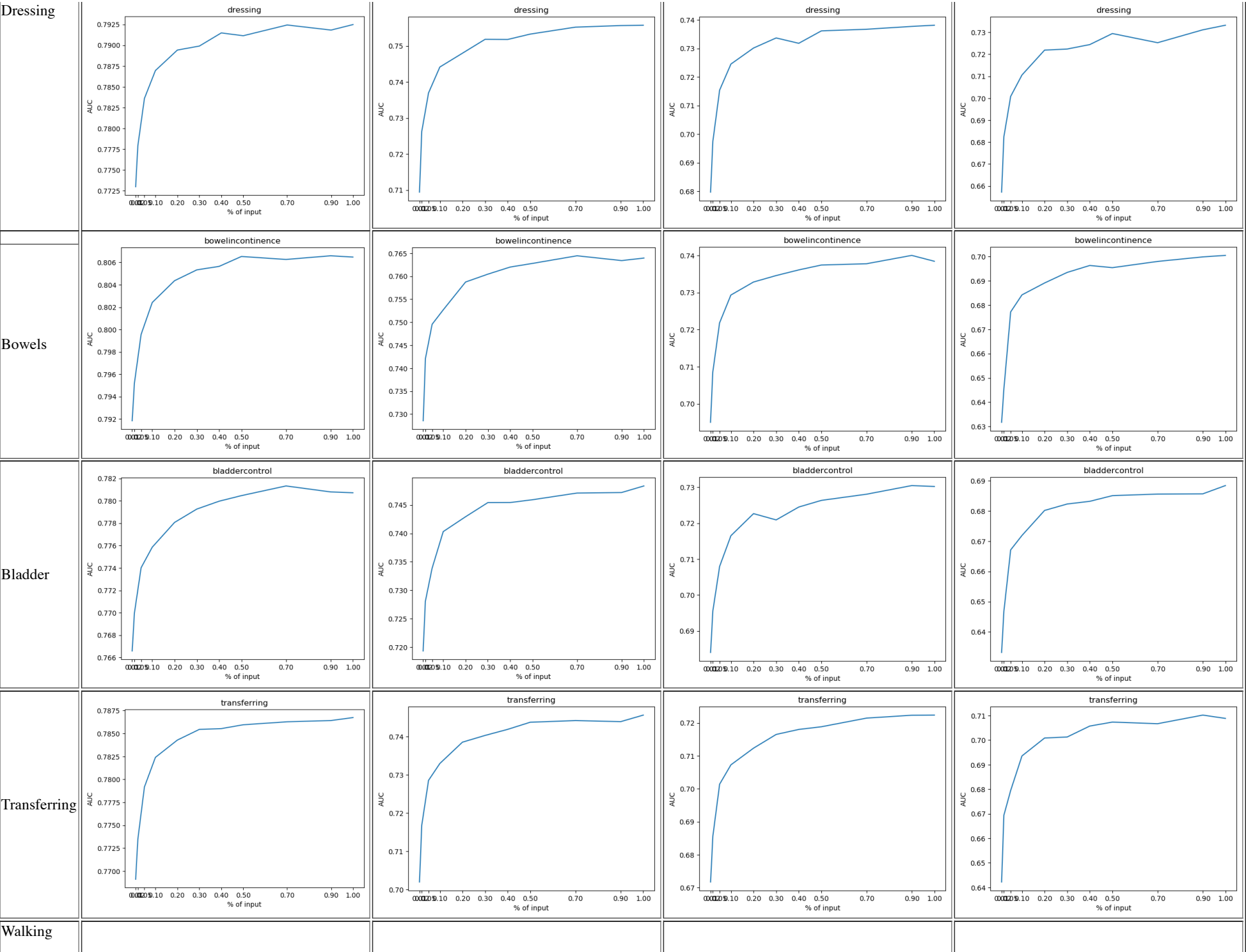

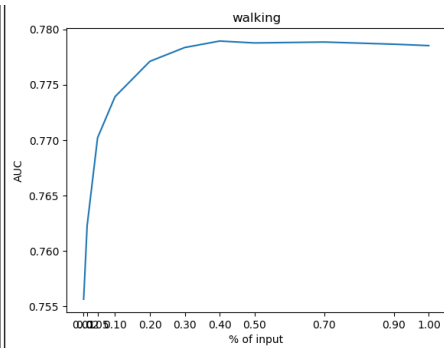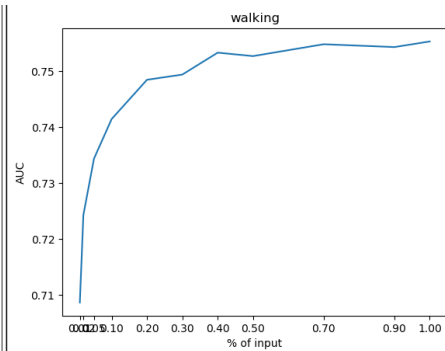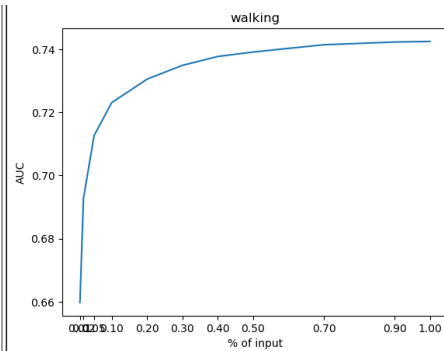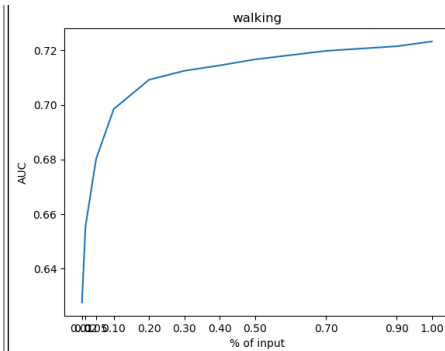

Supplement: Supplementary file 4 — Additional file 4. Learning_Curves_Full_Evaluation_Models. The file includes 36 learning curves for Full Evaluation Models in CBIT. [file 12911_2020_1368_MOESM4_ESM.pdf]

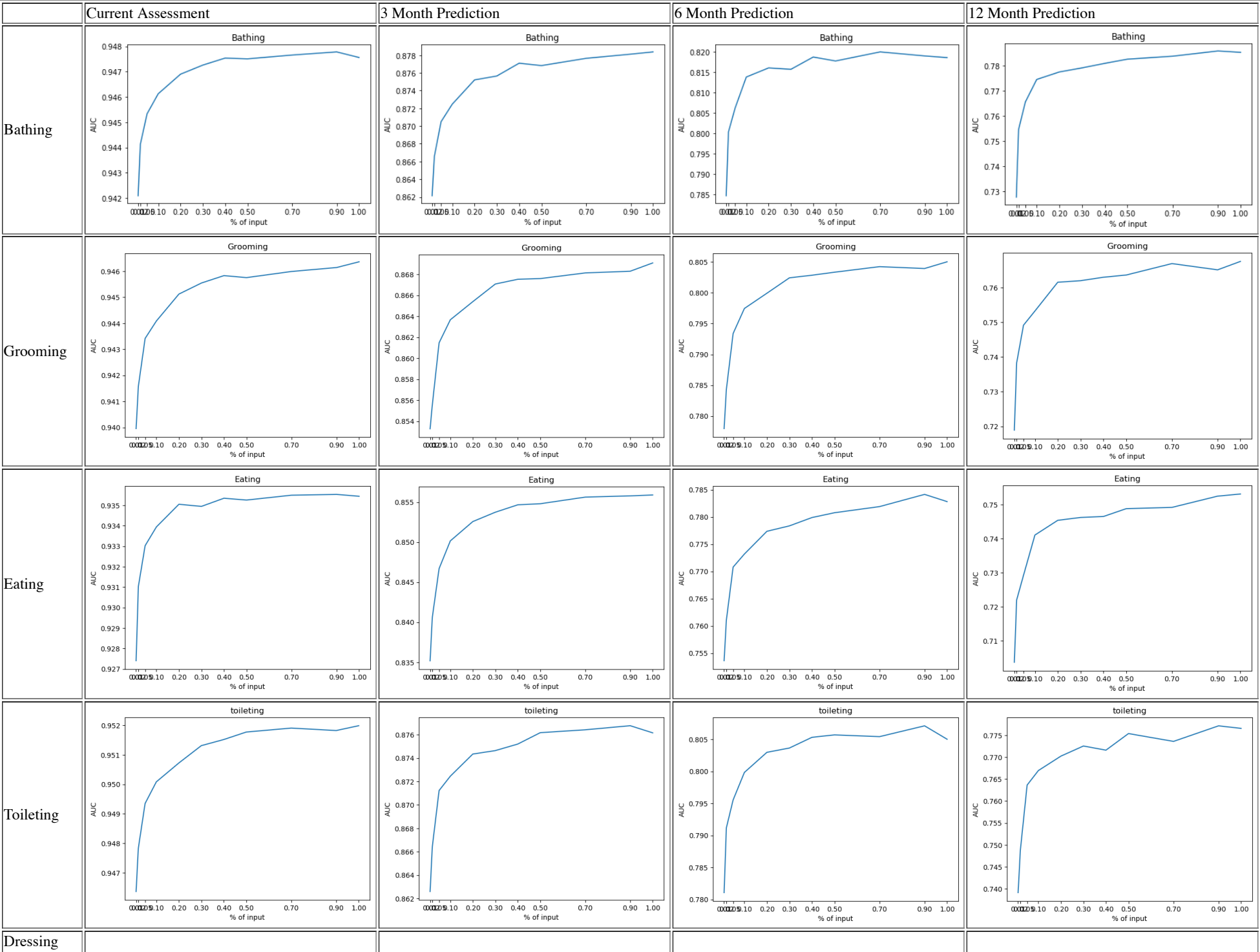

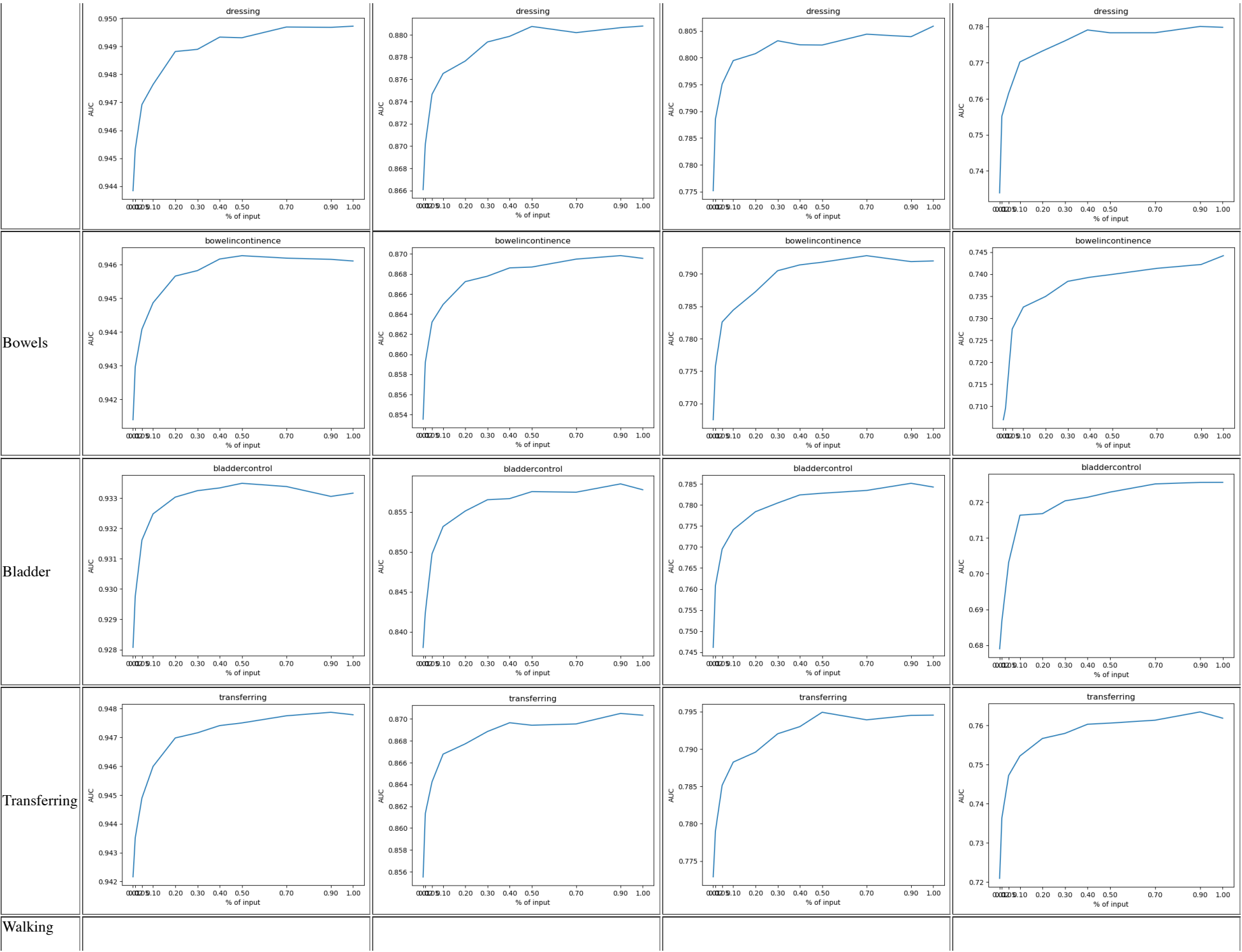

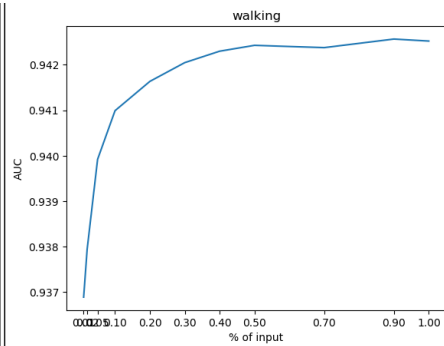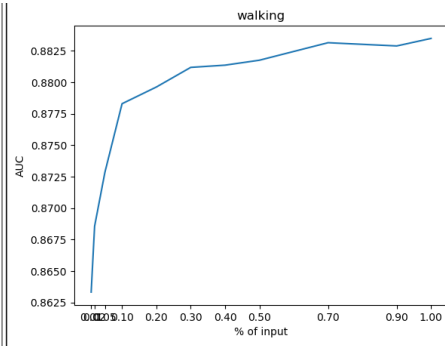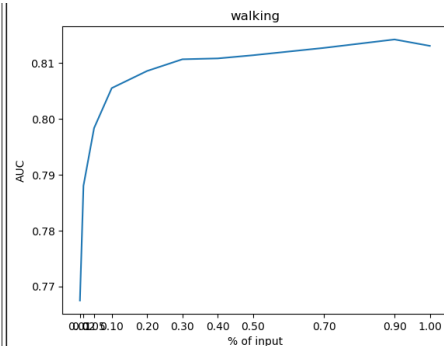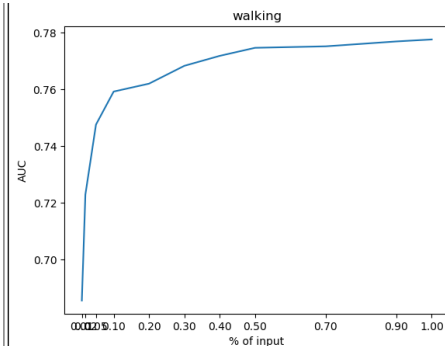

Supplement: Supplementary file 5 — Additional file 5. Learning_Curves_Full_Re-Evaluation_Models. The file includes 36 learning curves for Full Re-Evaluation Models in CBIT. [file 12911_2020_1368_MOESM5_ESM.pdf]

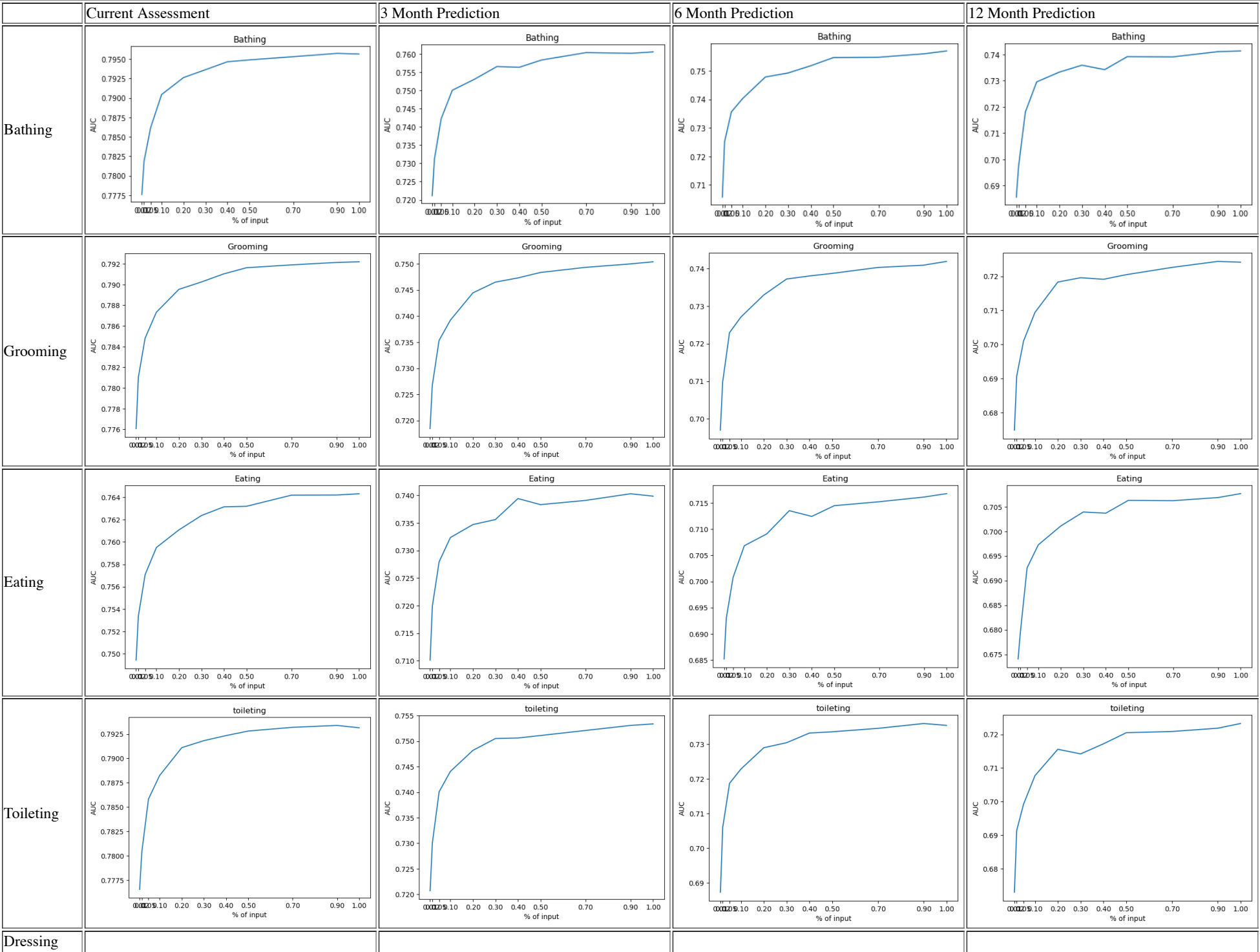

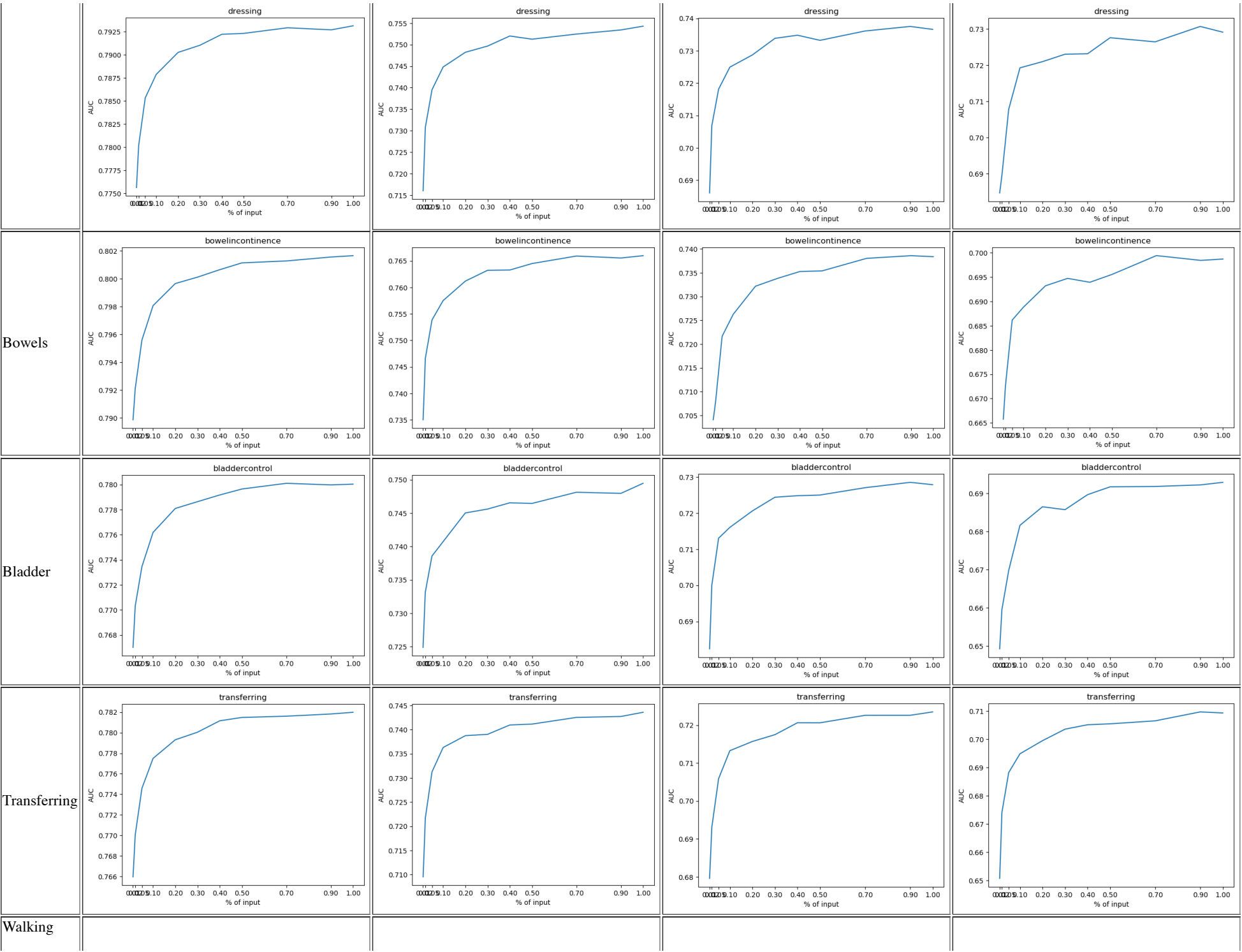

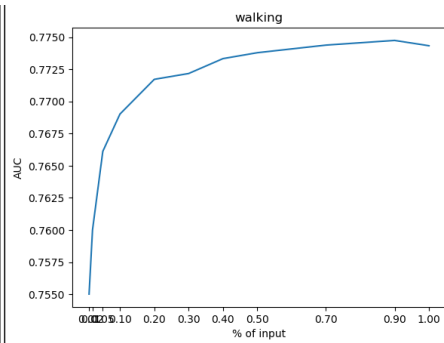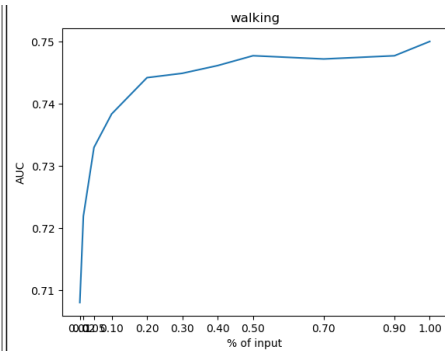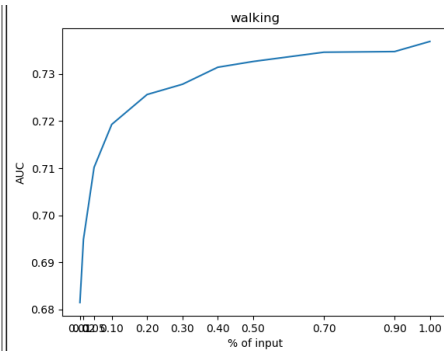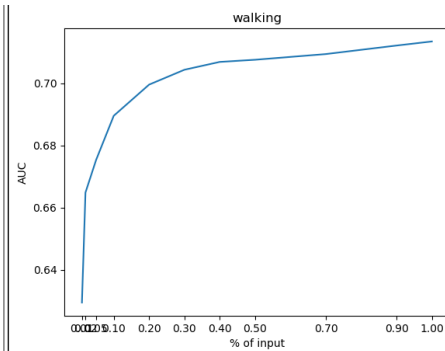

Supplement: Supplementary file 6 — Additional file 6. Learning_Curves_Simplified_Evaluation_Models. The file includes 36 learning curves for Simplified Evaluation Models in CBIT. [file 12911_2020_1368_MOESM6_ESM.pdf]

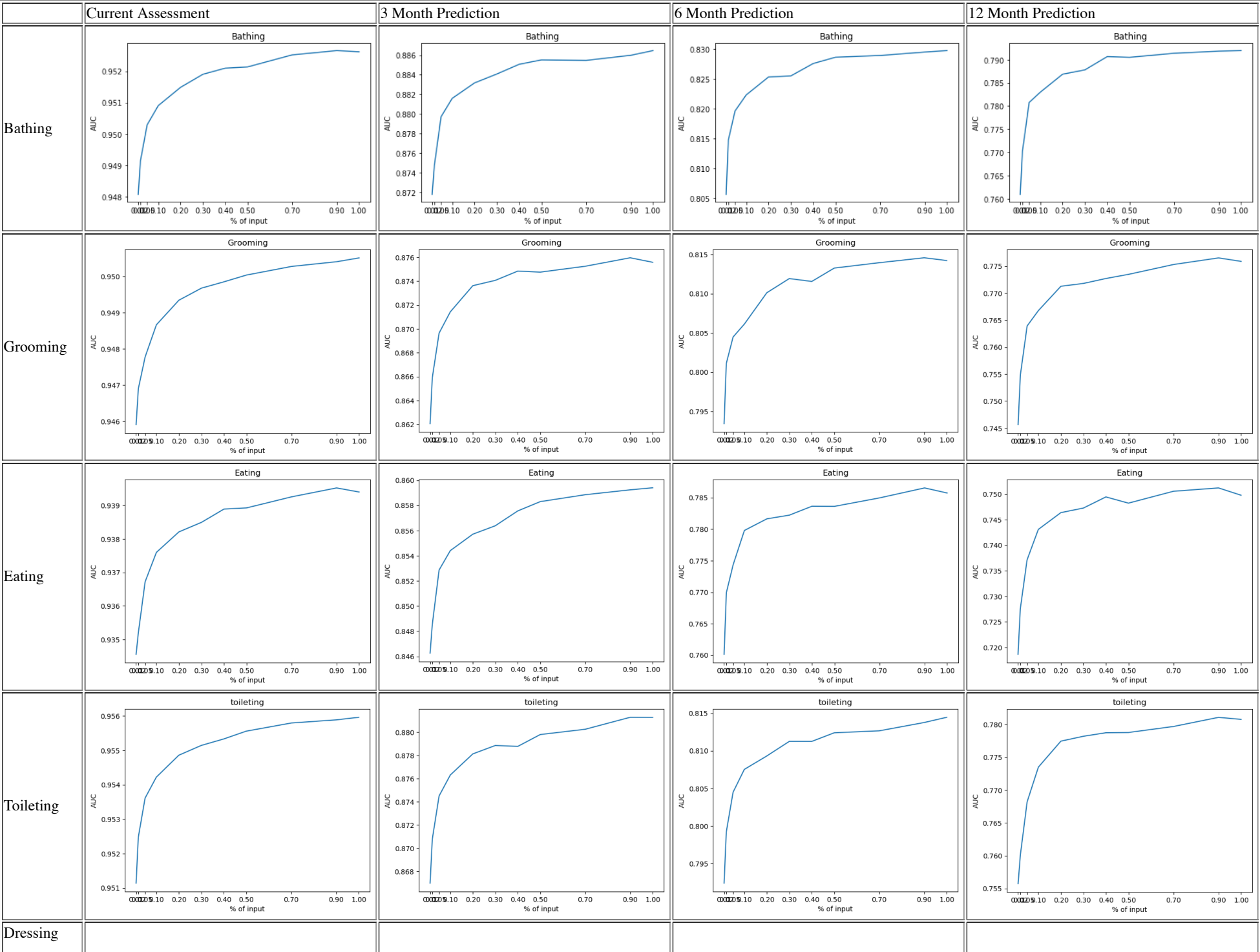

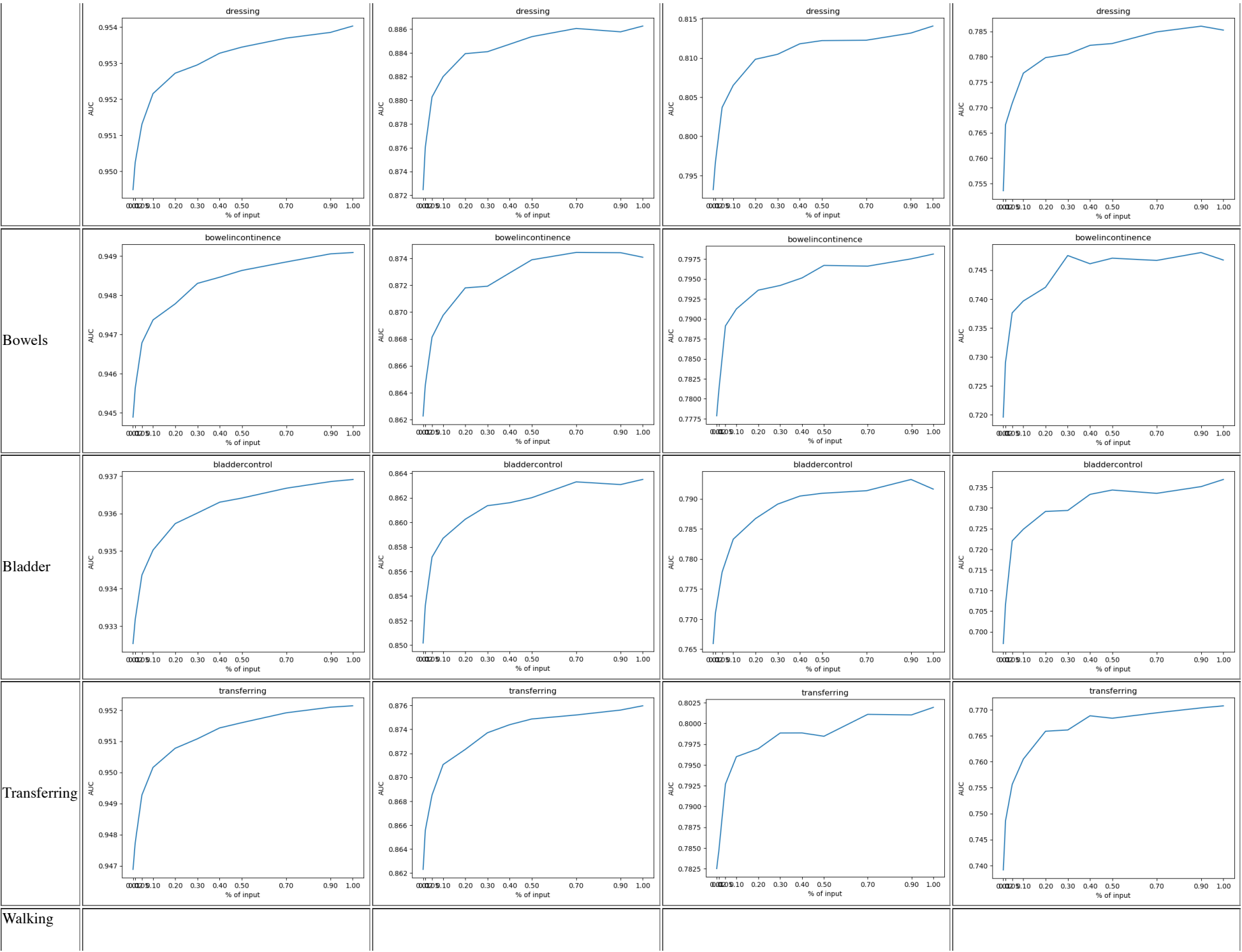

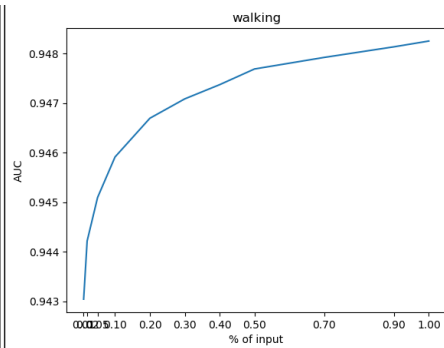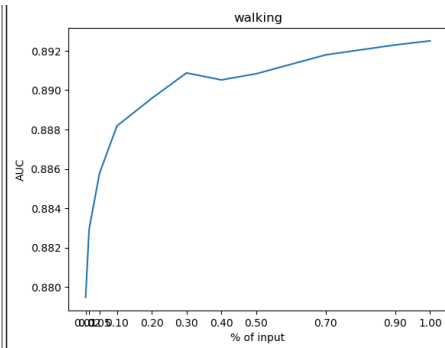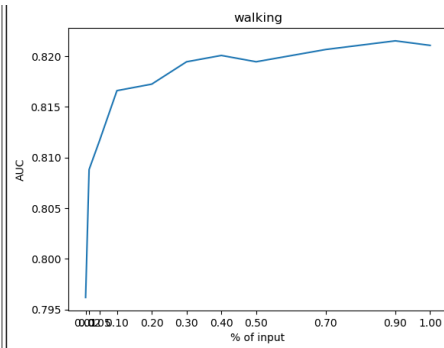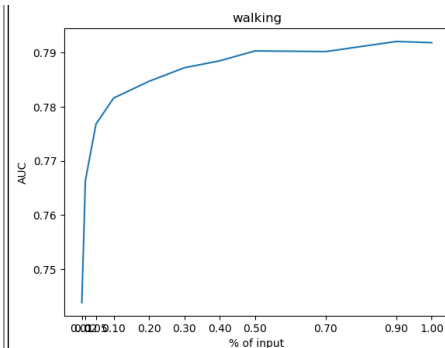

Supplement: Supplementary file 7 — Additional file 7. Learning_Curves_Simplified_Re-Evaluation_Models. The file includes 36 learning curves for Simplified Re-Evaluation Models in CBIT. [file 12911_2020_1368_MOESM7_ESM.pdf]

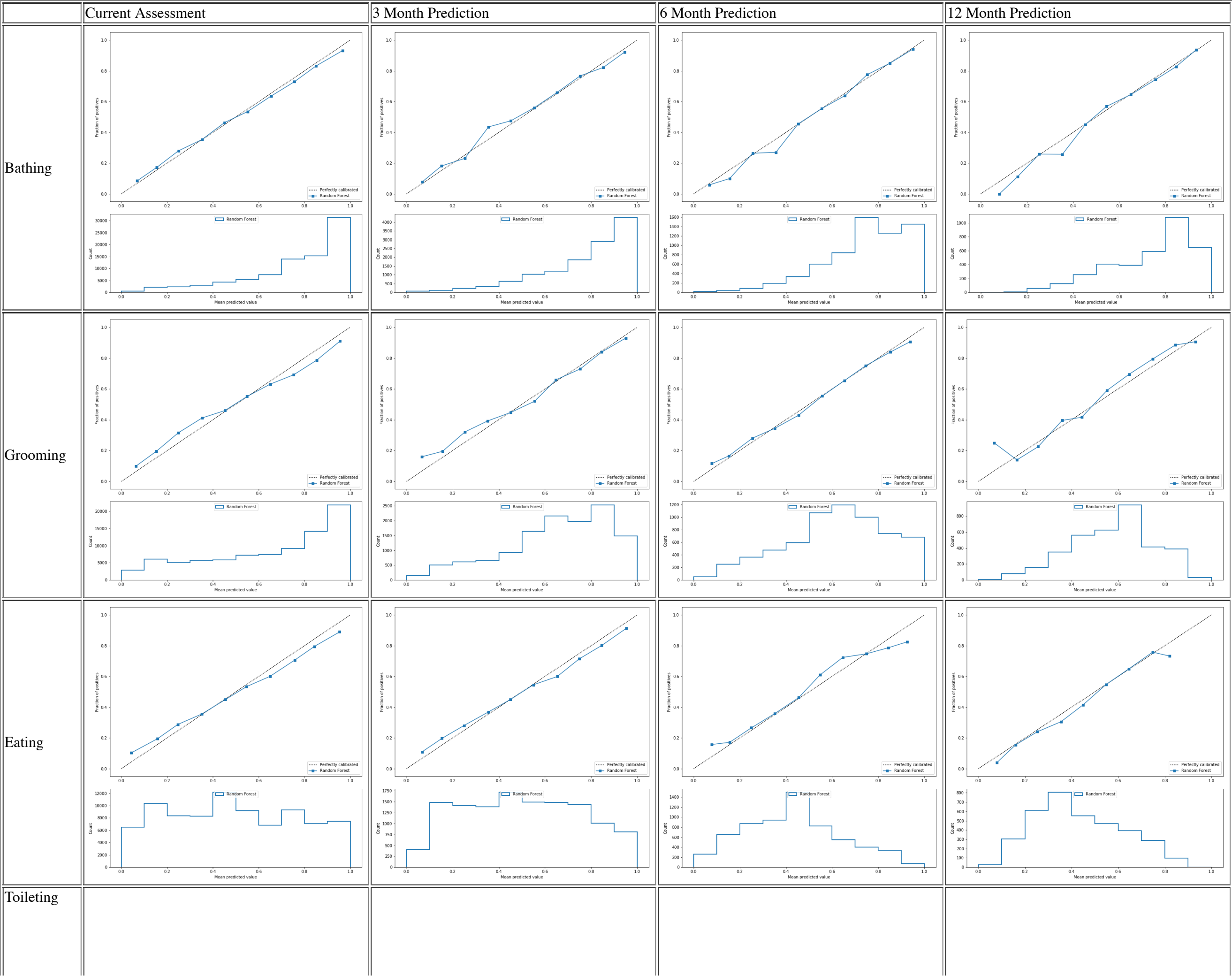

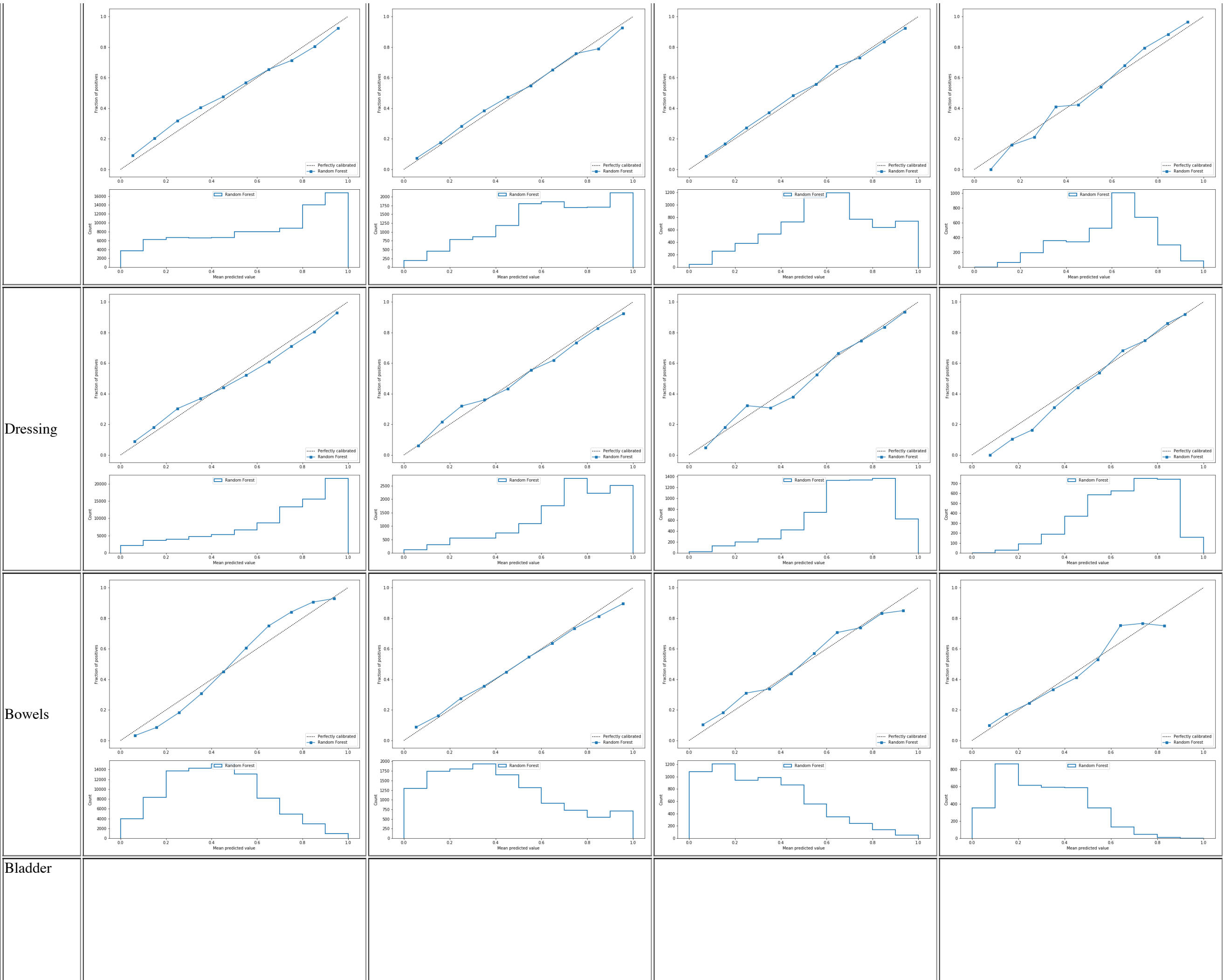

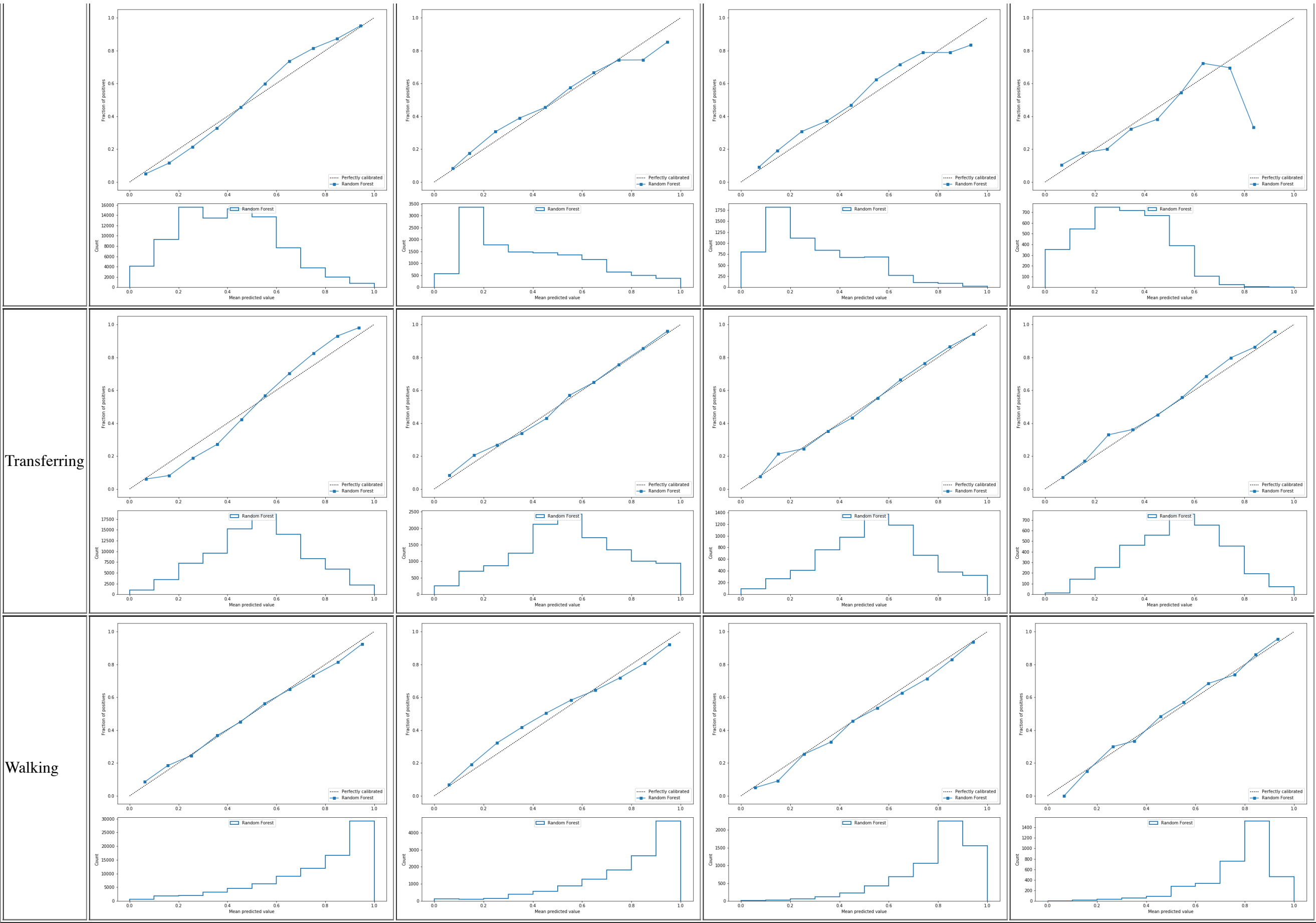

Supplement: Supplementary file 8 — Additional file 8. Calibration_Plots_Full_Evaluation_Models. The file includes 36 calibration plots for Full Evaluation Models in CBIT. [file 12911_2020_1368_MOESM8_ESM.pdf]

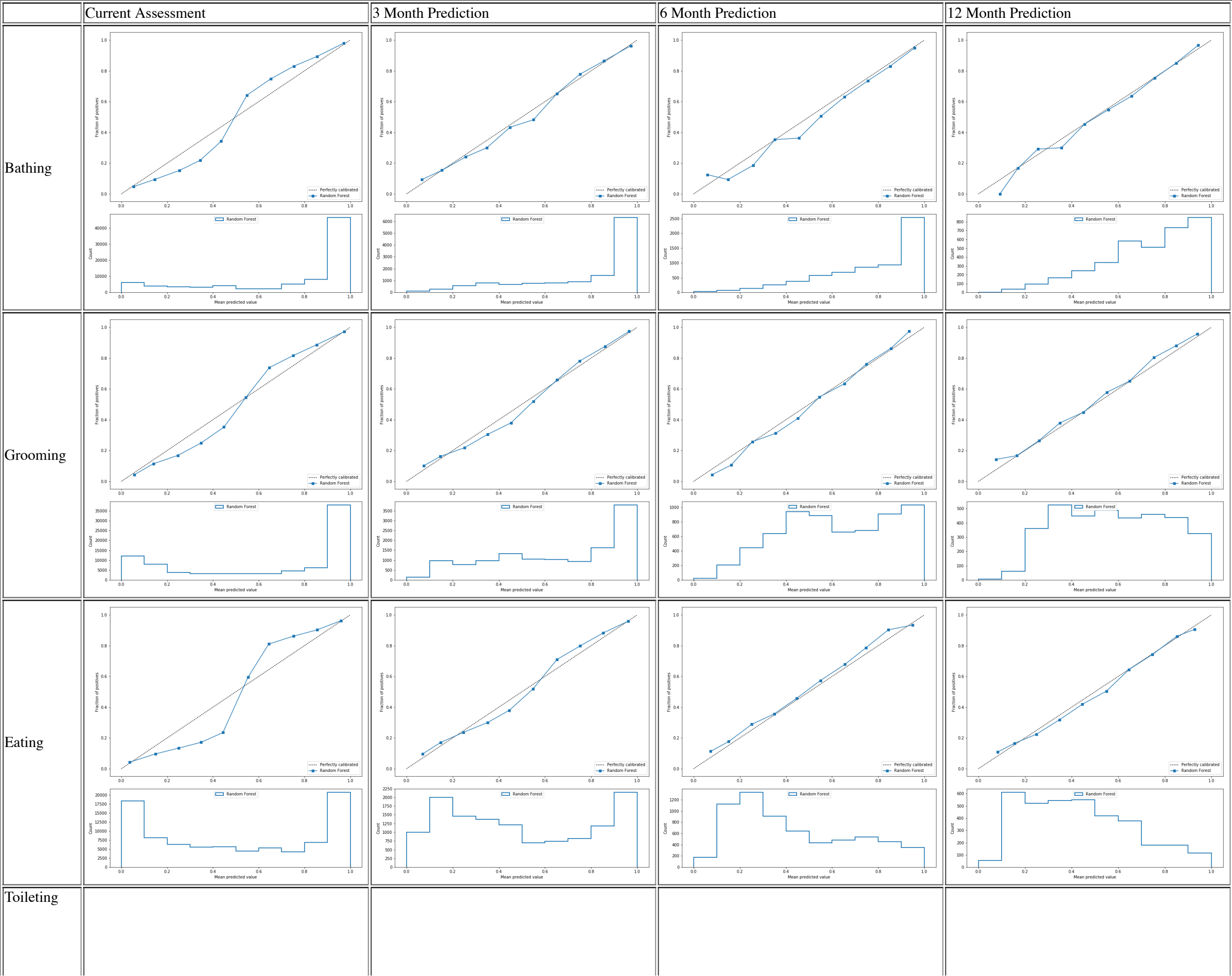

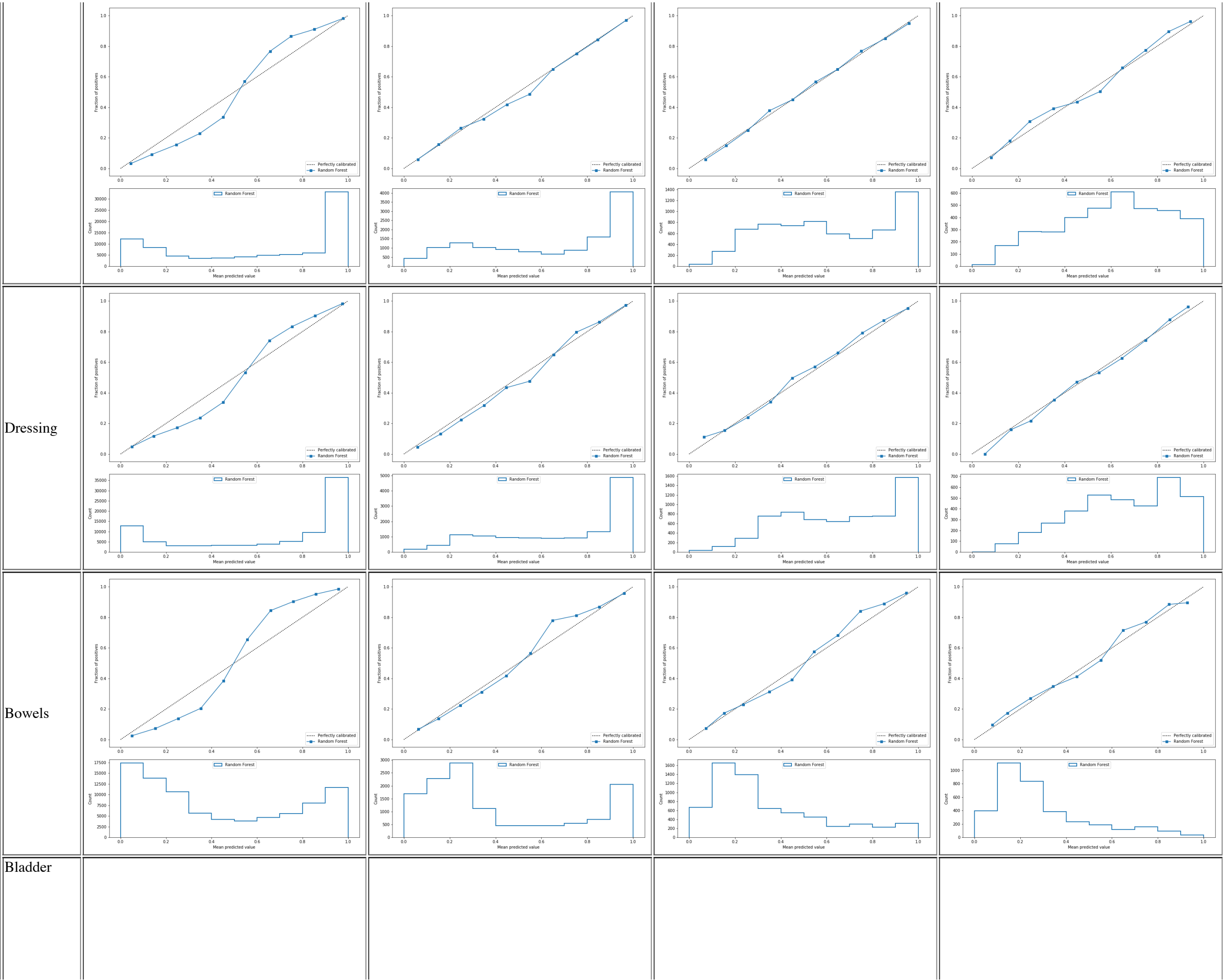

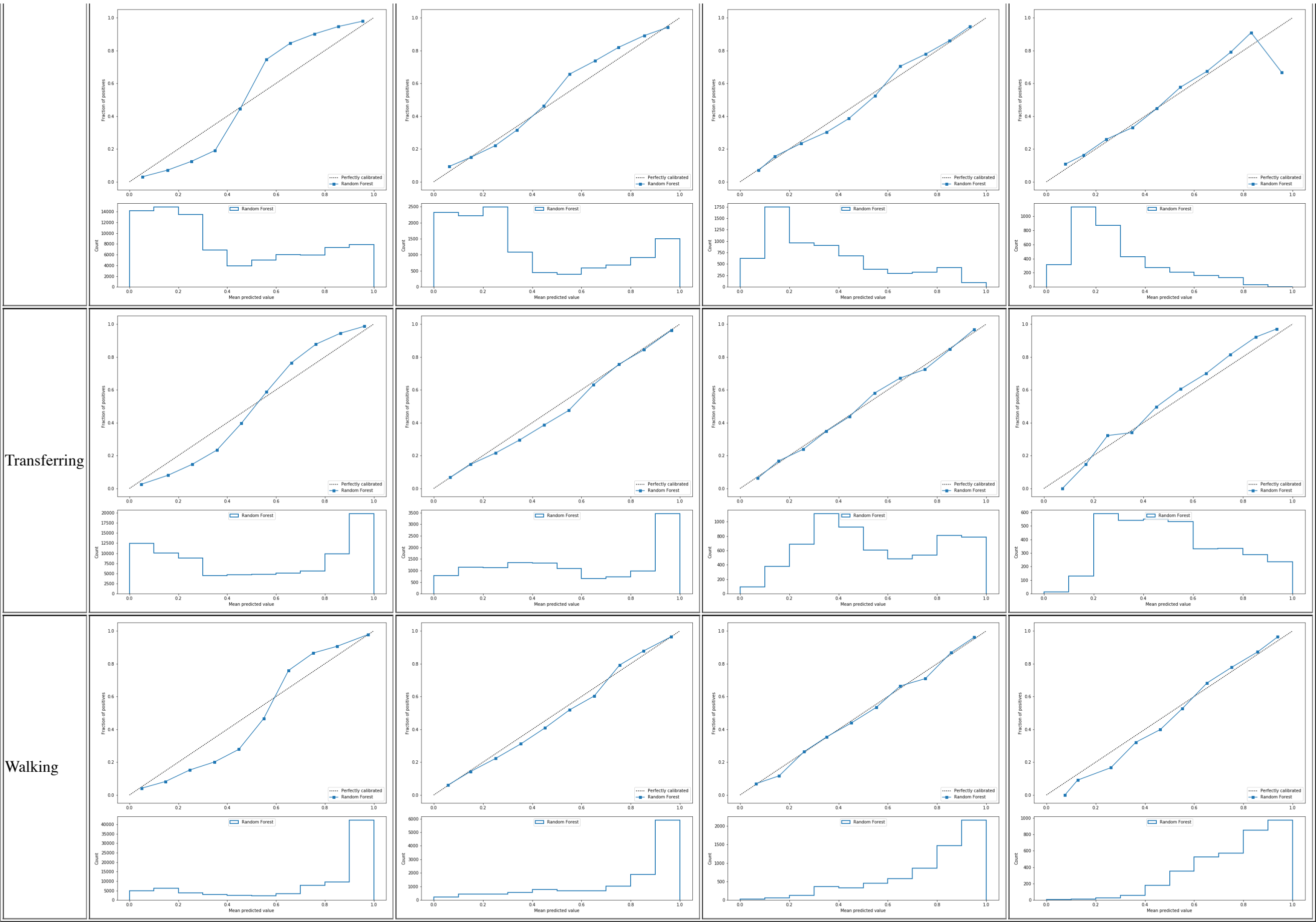

Supplement: Supplementary file 9 — Additional file 9. Calibration_Plots_Full_Re-Evaluation_Models. The file includes 36 calibration plots for Full Re-Evaluation Models in CBIT. [file 12911_2020_1368_MOESM9_ESM.pdf]

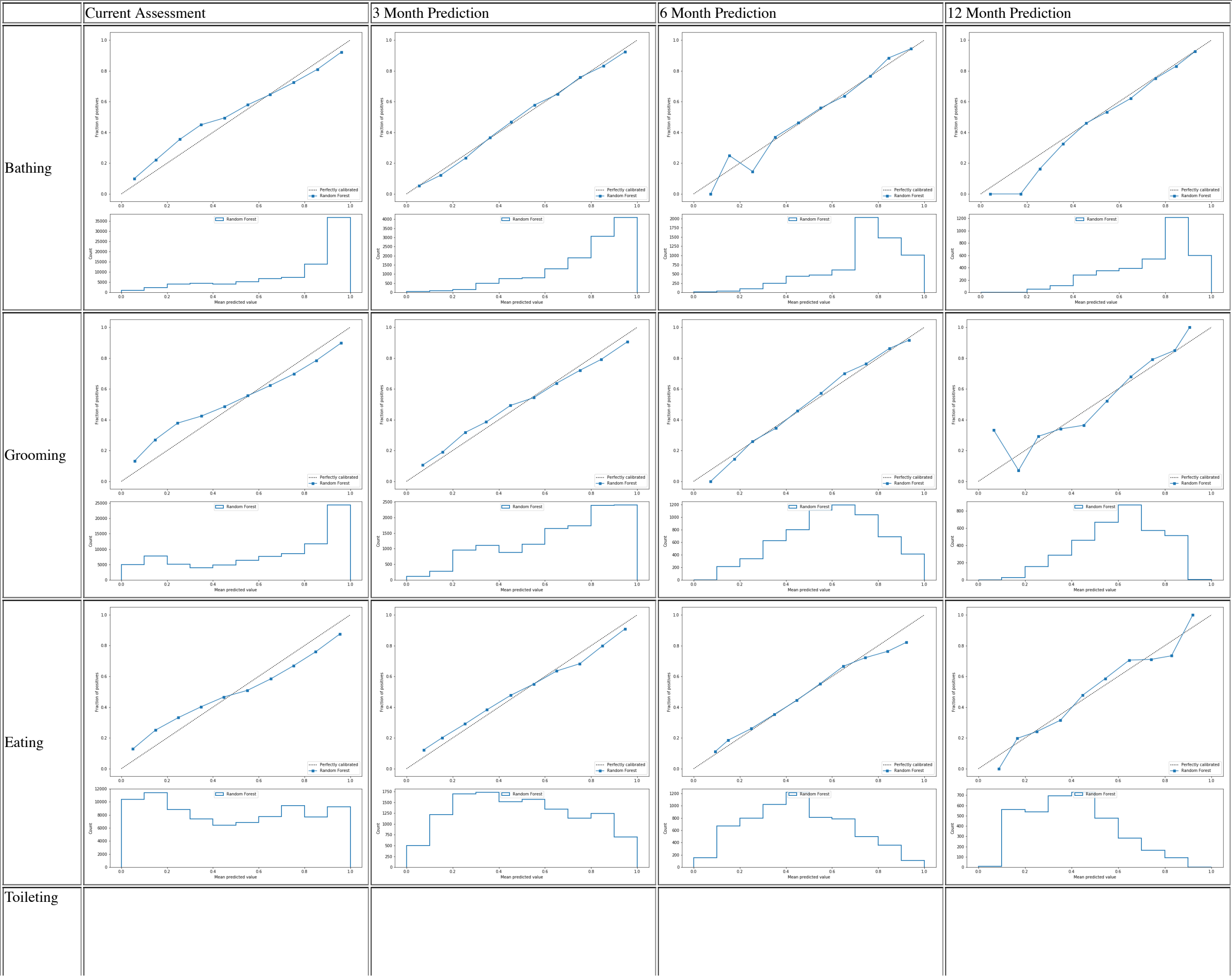

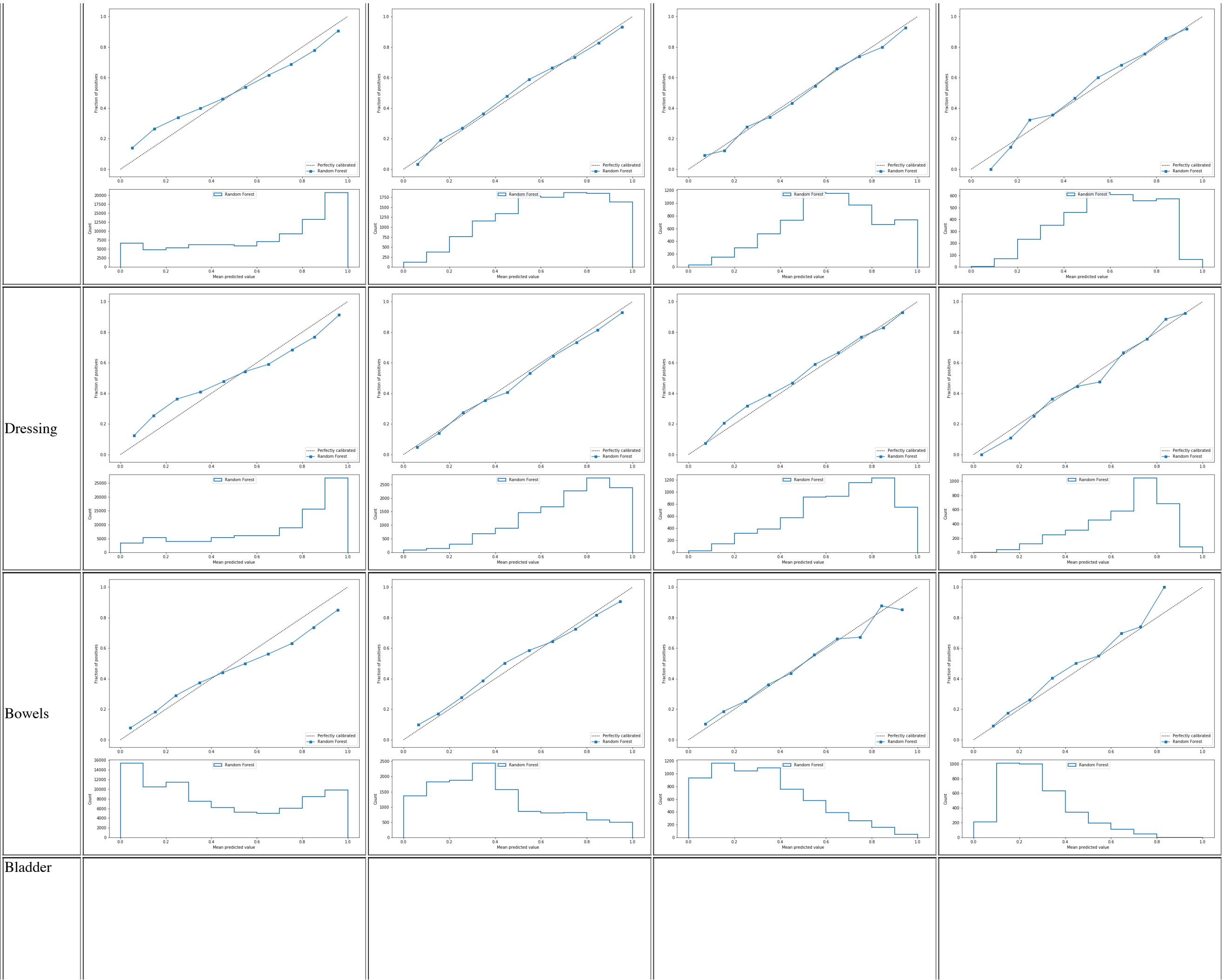

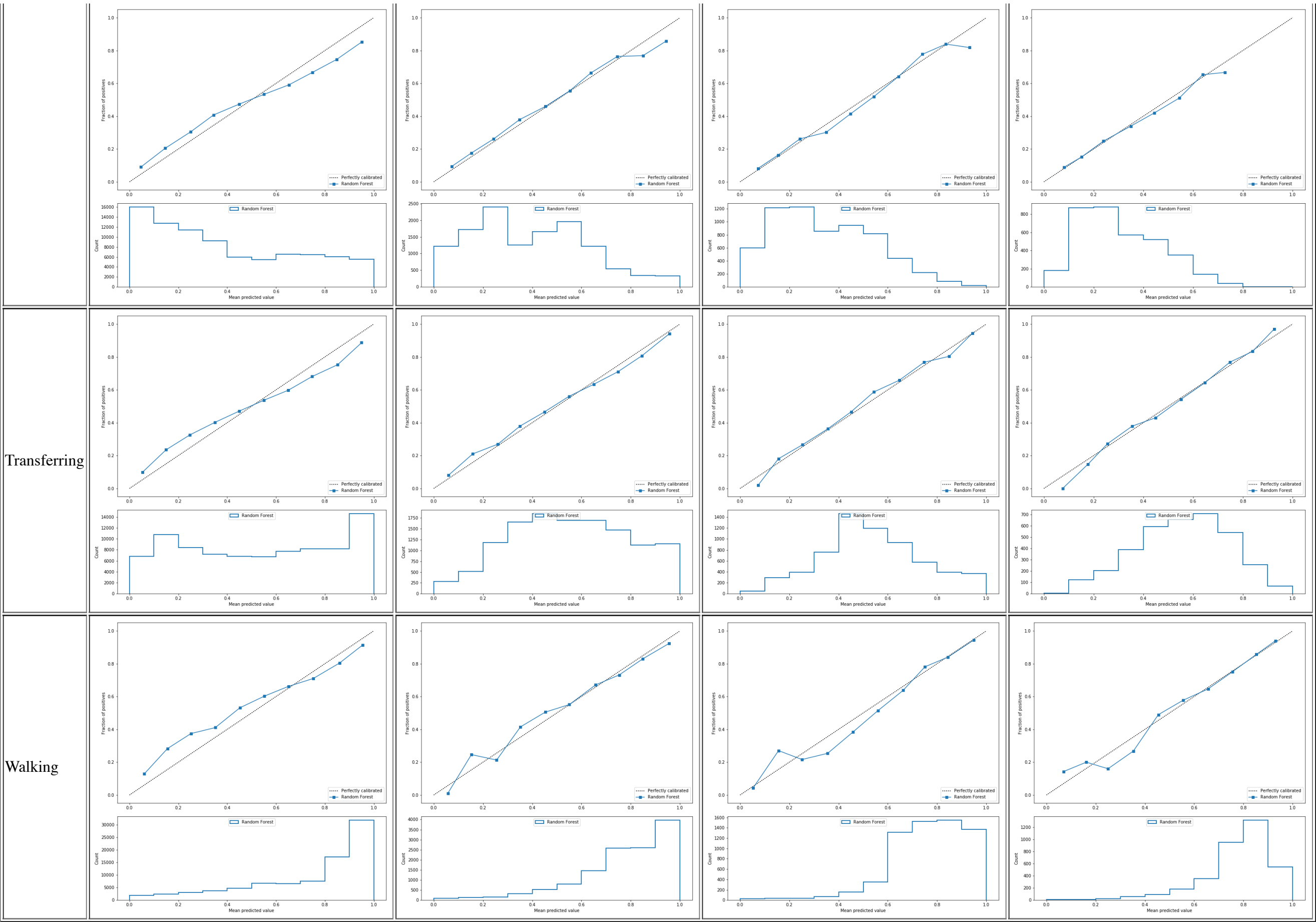

Supplement: Supplementary file 10 — Additional file 10. Calibration_Plots_Simplified_Evaluation_Models. The file includes 36 calibration plots for Simplified Evaluation Models in CBIT. [file 12911_2020_1368_MOESM10_ESM.pdf]

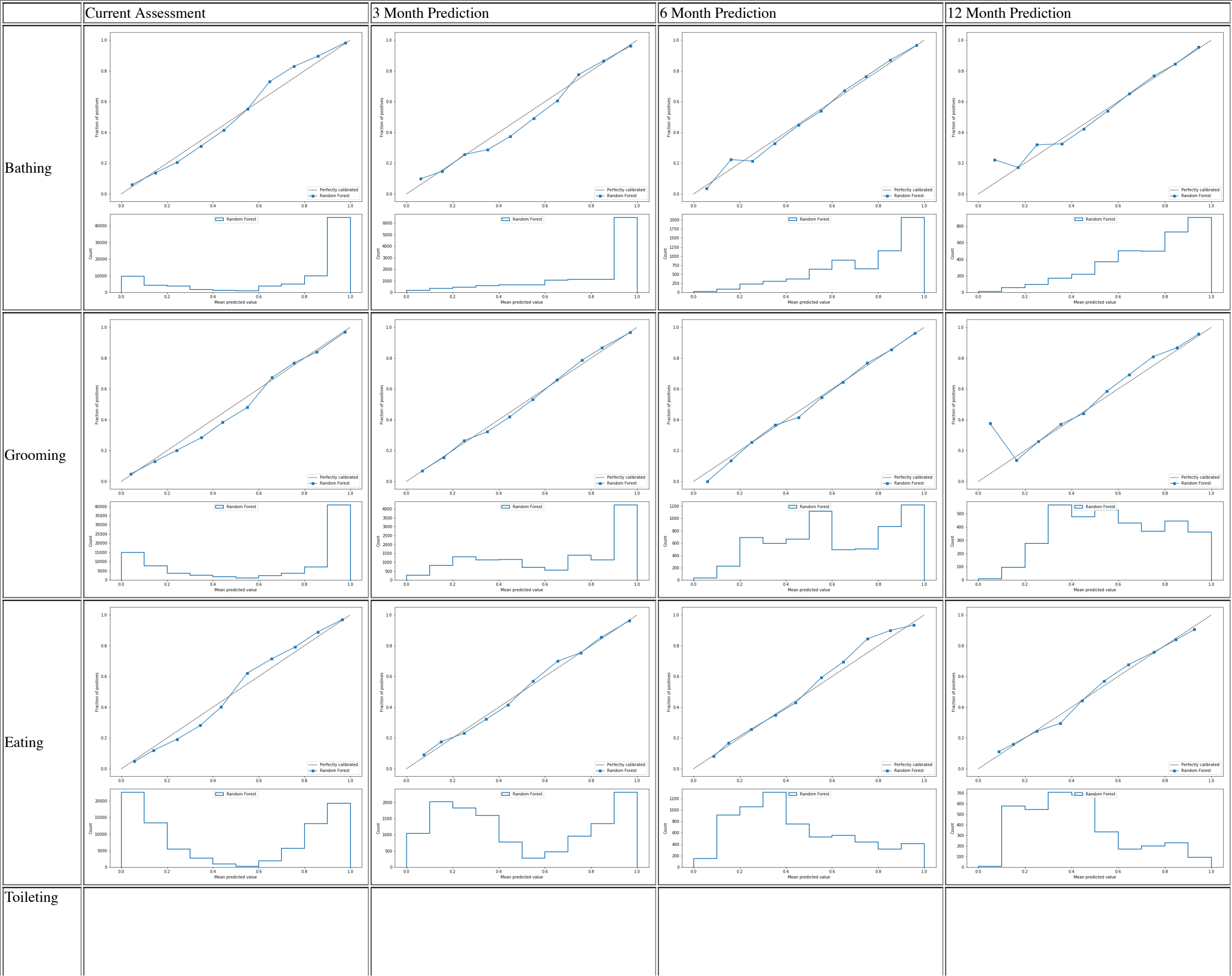

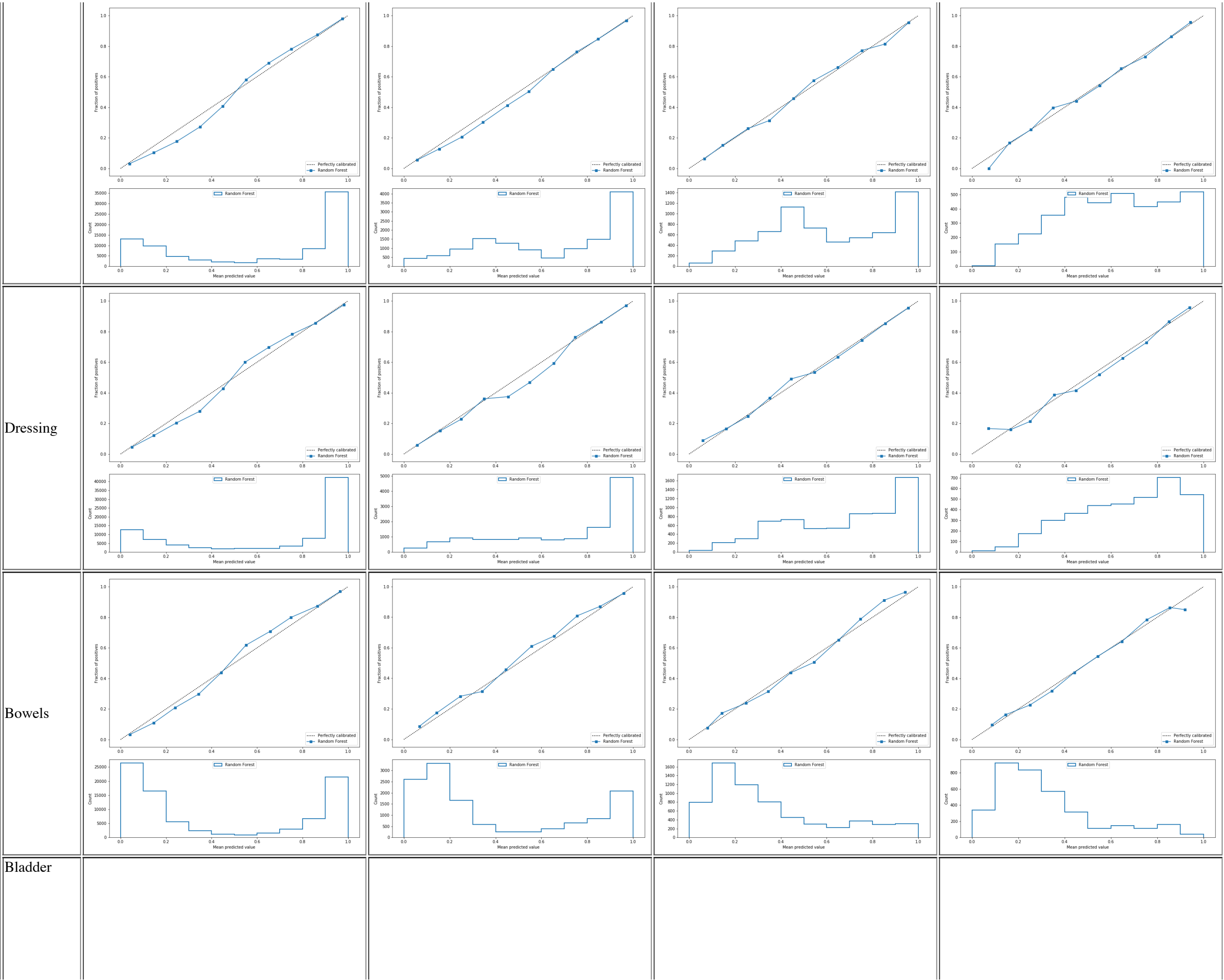

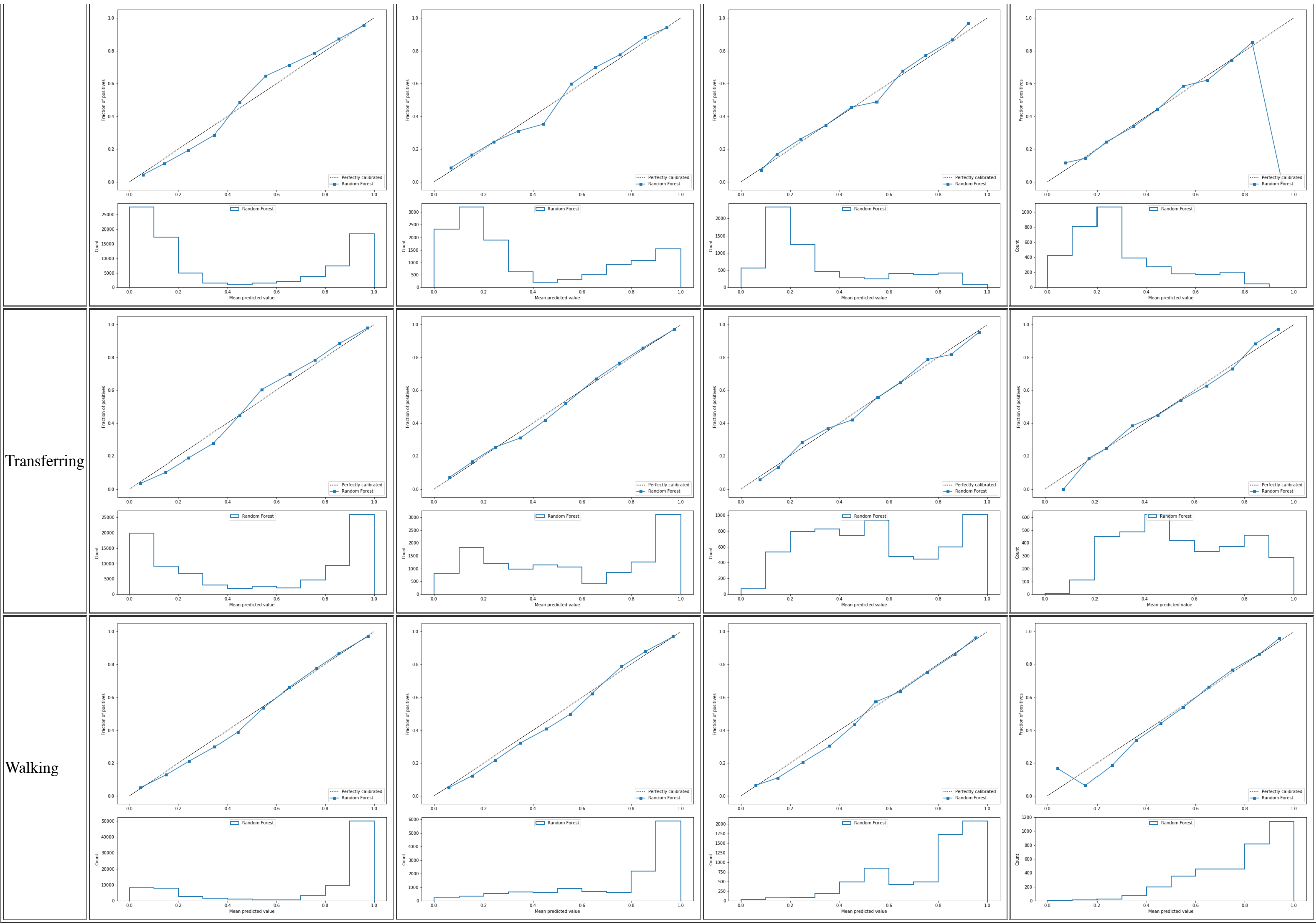

Supplement: Supplementary file 11 — Additional file 11. Calibration_Plots_Simplified_Re-Evaluation_Models. The file includes 36 calibration plots for Simplified Re-Evaluation Models in CBIT. [file 12911_2020_1368_MOESM11_ESM.pdf]
